# The comprehensive transcriptomic atlas of porcine immune tissues and the peripheral blood mononuclear cell (PBMC) immune dynamics reveal core immune genes

**DOI:** 10.1186/s40104-025-01184-y

**Published:** 2025-05-19

**Authors:** Qingyao Zhao, Jiahao Wang, Fuping Ma, Quanzhen Chen, Huatao Liu, Jinyan Yang, Siqian Chen, Yongjie Tang, Siyuan Mi, Lulu Wang, Xini Wang, Guohong Liu, Kai Xing, Ying Yu, Chuduan Wang

**Affiliations:** State Key Laboratory of Animal Biotech Breeding, National Engineering Laboratory for Animal Breeding, and Key Laboratory of Animal Genetics, Breeding and Reproduction, Ministry of Agriculture & College of Animal Science and Technology, China Agricultural University, Beijing, 100193 P. R. China

**Keywords:** Antiviral response, Core immune genes, Immune tissues, PBMC, Poly(I:C)

## Abstract

**Background:**

Viral diseases have profoundly influenced the sustainable development of the swine farming industry. With the development of genomics technology, the combination of transcriptome, genetic variation, immune response, and QTL mapping data to illustrate the interactions between pathogen and host immune system, will be an effective tool for identification of disease resistance genes in pigs. The immune system of an organism is the source of disease resistance in livestock, consisting of various immune tissues, as well as the immune cells and cytokines they produced. However, comprehensive systematic studies on transcriptome of porcine immune tissues are still rare. Poly(I:C), as a viral mimic, is commonly used to study immune responses of the body during viral infections, and serves as a valuable tool for investigating immune mechanisms in swine.

**Results:**

WGCNA analysis identified core immune genes across six immune tissues (bone marrow, jejunum, lymph node, PBMC, spleen, thymus) in Landrace pigs, which are also crucial for the development of PBMCs. The examination of the changes in the proportion of immune cells during three developmental stages (1-month-old, 4-month-old, 7-month-old) shows a shift from innate immunity to humoral immunity. By integrating different epigenetic genomics datasets, we identified several core immune genes and their causal variants, including *IFI44*, *IFIT5*, *EIF2AK2* and others, which are closely related to immune development and response. Functional validation studies reveal that the *IFI44* gene acts as a negative regulator of the antiviral response; its inhibition effect significantly reduced Poly(I:C)-induced cell necrosis, while enhancing apoptosis to combat viral infections.

**Conclusion:**

Our study elucidated the fundamental transcriptional program in porcine immune tissues and the immunodynamics underlying development of PBMCs, identifying many core immune genes, including *IFI44*, which plays a critical negative regulator role in the antiviral response, providing valuable insights for breeding programs aimed at enhancing pig disease resistance.

**Supplementary Information:**

The online version contains supplementary material available at 10.1186/s40104-025-01184-y.

## Background

Pork is one of the most widely consumed meats globally, accounting for around a third of the world’s total meat production and having significant impacts on the diets and economies of many countries, with nearly half of the world’s total output coming from China [1]. However, infectious diseases are a major global constraint to porcine production, and local or emerging pathogens are extremely easy to quickly spread around the world, resulting in serious consequences and huge economic losses [2]. Over the past few decades, genetic selection of modern commercial pig breeds has focused mainly on production traits and meat quality improvement, which can lead to reduced or missing expression of certain immune-related genes, resulting in inadequate immunity and making them susceptible to infection under intensive farming conditions, thus hinders normal productivity [3, 4]. In recent years, through genome-wide association studies, a large number of genetic variations associated with complex traits in pigs have been uncovered [5, 6]. However, progress in exploring porcine genetic variations and resistance genes related to immunity and health has been slow due to the difficulty in measuring disease resistance phenotypes. The most successful example in pigs is the discovery of the *CD163* gene, which confers resistant to porcine reproductive and respiratory syndrome virus (PRRSV), as revealed through functional genomics and virus-host interaction studies [7, 8], demonstrating that different types of genomic tools can complement each other and jointly promote immune and disease research.

The Farm Genotype-Tissue Expression (FarmGTEx) project has demonstrated that exploring the gene expression of different types of tissues can help unravel the genetic mechanisms behind their complex phenotypes [9, 10]. By comparing different type of muscle and adipose tissues in pigs, Jin et al. discovered *ACE2* gene associated with obesity [11], which is a crucial therapeutic target for diabetes in humans [12], and Tkatchenko et al. discovered the *CMYA5* gene associated with Duchenne muscular dystrophy [13]. The immune system of an organism is the most effective weapon against external pathogens and is the source of disease resistance in pigs. It consists of various immune tissues, as well as the immune cells and cytokines they produce [14–16]. Although gene expression data has been collected for 34 different tissues in pigs in previous studies [10], the research lacks specificity and may lead to inadequate mining of patterns between tissue types with specific functions. Furthermore, comprehensive systematic studies on porcine immune tissues are still rare.

Here, by comprehensively collecting six porcine representative immune tissues (bone marrow, thymus, spleen, lymph node, blood, small intestine), we systematically investigated and functionally characterized the immune tissue transcriptome landscapes, and explored the dynamics of porcine peripheral blood mononuclear cells (PBMCs) across the three developmental stages of the immune system. In addition, we annotated the changes in the proportion of immune cells in porcine PBMCs during development by integrating multi-omics datasets of eight distinct immune cell types isolated from PBMCs, including histone modifications, gene expression, and chromatin accessibility. Due to genetic differences in immunity between breeds [17–19], we also compared the differences in immune cell proportions between different breeds. By detecting common patterns of gene expression among immune tissues, we identified core immune genes and potential causal variants by combining Hi-C datasets of pig fibroblasts with multi-omics data from PBMCs. Finally, through cellular assays, we validated the negative regulatory function of *IFI44*, a core immune gene, in the antiviral response. Overall, our study revealed the core transcriptional programs associated with pig immune function and provided candidate genes and potential causal variants in pig immune phenotypes.

## Methods

### Experimental animals and sample collection

Five healthy male Landrace piglets were selected from a commercial farm in Beijing, with an average age of 37.6 ± 2.14 d (mean ± SD) and an average weaning age of 26.8 d. The five piglets were sent to the Animal Medicine Laboratory of China Agricultural University for slaughter and sampling. All animal procedures were performed according to protocols of the Institutional Animal Care and Use Committee (IACUC) at China Agricultural University. Briefly, the peripheral blood, spleen, cervical lymph node, thymus, small intestine (jejunum segment) and bone marrow (extract from the left femoral joint using a bone marrow puncture needle) were collected immediately after the animals were sacrificed. The tissue samples except blood were quickly frozen in liquid nitrogen, and PBMCs were separated from peripheral blood using the porcine peripheral blood mononuclear cell isolation solution kit (Solarbio Science & Technology Co., Ltd., Beijing, China) within 4 h, and stored at −80 °C. Additionally, five 4-month-old (average 126.6 ± 2.16 d) and five 7-month-old (average 208.6 ± 20.16 d) healthy male Landrace pigs, as well as five adult healthy male Songliao Black pigs (1–3 years), were selected from the same commercial farm for peripheral blood collection. As above, PBMCs were isolated within 4 h and stored at −80 °C.

### RNA isolation and transcriptomic sequencing

The total RNA was extracted from porcine immune tissue samples by TRIzol reagent (Invitrogen, Carlsbad, CA, USA) according to the manufacturer’s instruction. The KaiaoK5500® Spectrophotometer (Kaiao, Beijing, China) was adopted to monitor RNA purity. The concentrations of isolated RNA were determined using the NanoDrop spectrophotometer. RNA integrity and concentration were assessed using the RNA Nano 6000 Assay Kit on the Bioanalyzer 2100 system (Agilent Technologies, CA, USA). RNA degradation and contamination were assessed using 1% agarose gel electrophoresis. Then the qualified total RNA from each sample were sent to (BGI TechSolutions Co. Ltd., (Beijing, China) for sequencing. The RNA transcriptomic sequencing library was prepared using BGI standardized protocol involving mRNA enrichment with oligo(dT) magnetic beads, RNA fragmentation, cDNA synthesis, adapter ligation, and PCR amplification. The final products were circularized using rolling circle amplification to generate DNA nanoballs (DNBs), which were loaded onto patterned nanoarrays for pair-end sequencing on the DNBseq platform. After sequencing, raw reads were filtered by removing adaptor sequences, contamination and low-quality reads.

### Identification of tissue-specific expression genes

After quality control, the clean reads were mapped to the reference genome (Sscrofa11.1) using STAR software (v2.7.11a) [20]. The number of reads per gene was counted using featureCounts (v2.0.6) [21] based on ENSEMBL *Sus scrofa* gene annotation file (Sus_scrofa.Sscrofa11.1.106.gtf). The gene expression levels were determined by normalizing the read counts to transcripts per kilobase per million mapped reads (TPM). The genes with a threshold of TPM ≥ 0.1 in ≥ 20% of samples were defined as the expressed genes and used for subsequent analysis. Then, the tissue-specific index tau value (τ) of each gene expressed in six immune tissues of Landrace piglets was calculated [22], and the genes with τ value > 0.8 were selected as tissue-specific expression genes, and the genes with expression quantity in top 25% quantile were further defined as tissue-specific high expression genes.

### Differential expression and gene function enrichment analysis

The differential expression analysis of PBMCs and various tissues including thymus, jejunum, spleen, lymph, and bone marrow in Landrace piglets was conducted using the DESeq2 R package (1.34.0) based on the negative binomial distribution [23]. Additionally, differential expression of PBMCs at three different age stages (1-month-old, 4-month-old, 7-month-old) in Landrace pigs was analyzed. Furthermore, the differential expression of PBMCs in Songliao black pigs and Landrace pigs(7-month-old) was also investigated. The *P* value < 0.05 and |log_2_(Fold change)| > 1 were set as the threshold to define differentially expressed genes (DEGs).

We performed gene set enrichment analysis (GSEA) on the differentially expressed results of PBMCs and other five immune tissues in Landrace piglets using GSEABase R package (v1.64.0). Kyoto Encyclopedia of Genes and Genomes (KEGG, release 110.0) and Gene Ontology (GO, release 2024-01-17) pathway enrichment analysis for DEGs were performed using the clusterProfiler R package (v4.10.1). GO terms and KEGG pathway with *P* < 0.05 were considered to be significantly enriched. For GO pathway enrichment, only “Biological Process” GO terms were considered.

### Calculation of pathway activity scores across all samples

We used the R packages Gene set variation analysis (GSVA; v1.50.1) [24], which estimates variation of pathway activity, to calculate individual mean scores for several immune-related GO/KEGG pathways. The TPM expression matrix from all sample was used as input, and the pathway activity was evaluated in combination with the gene set extracted from each immune-related pathway of interest, and each pathway corresponding to each individual was able to obtain only one pathway activity score.

### Weighted correlation network analysis of six immune tissues in Landrace piglets

To identify the co-expression modules from expressed genes of six immune tissues in Landrace piglets, we performed a Weighted Correlation Network Analysis (WGCNA) using the WGCNA (v1.72) R package [25]. Briefly, the filtered TPM expression matrix of six immune tissues was used as input. The output was the gene modules according to their expression patterns, and an optimal soft-thresholding power of eight (Scale-free *R*^2 ^= 0.84) was selected to ensure a scale-free topology. The module eigengene (MEs, the first principal component of the module) was chosen to represent the expression pattern. Then the immune pathway activity scores of 30 samples of six immune tissues were used as phenotypes, and correlation analysis was conducted with the obtained co-expression modules to find the key modules. The module membership (MM) score was calculated for the genes in the key modules to find the hub genes with the highest connectivity.

### Time-series clustering and enrichment analysis

In order to explore the gene expression pattern of PBMCs with age change, we conducted time series analysis on PBMC data of three different periods using Mfuzz (v2.62.0) R package [26]. The TPM average expression data from PBMCs across three distinct time periods were utilized as input, with the cluster number set to 16. As a result, we obtained 16 gene clusters exhibiting diverse expression patterns over time. Subsequently, core genes were extracted from each cluster based on a minimum membership threshold (acore > 0.5). The core genes of the clusters whose expression pattern is up-regulated with age were merged and defined as the up-regulated cluster, while the core genes of the clusters whose expression pattern is down-regulated with age were merged and defined as the down-regulated cluster. The gene clusters with “V-shaped” trend were excluded from our consideration. Pathway enrichment analysis was performed for up-regulated and down-regulated gene clusters.

### Deconvolution analysis of PBMC types

We obtained RNA-seq data of purified cells from the National Center for Biotechnology Information Sequence Read Archive (NCBI SRA) database (PRJEB43826), which includes eight distinct immune cell types (CD4αβT, CD4 helper T, CD8 T, gamma delta T, myeloid leukocyte, memory B, naïve B, NK) isolated from two porcine PBMCs using Fluidic sorting. The TPM expression matrix of each cell type was obtained through the above transcriptome analysis process. The gene expression matrix of the eight cell types was used to generate a reference file for uploading to CIBERSORTx [27]. The gene expression matrix of PBMCs in three different periods was used as a cell mixture to generate the corresponding mixture file.

The CIBERSORTx signature matrix consisting of ‘barcode’ gene was generated from the reference file using default CIBERSORTx parameters. To impute the fraction of cell types within the cellular mixtures, CIBERSORTx was run using 1,000 permutations.

### SNP calling and association analysis

According to the pigGTEx-Pipeline [10] process, we get single nucleotide polymorphism (SNP) information from the transcriptome data. In brief, Picard tools were utilized to incorporate read groups, sort, mark duplicates, and index the reads into the bam file for each PBMC sample. GATK’s SplitNCigarReads was employed to handle reads spanning introns and adjust mapping qualities [28]. Base quality scores were recalibrated using GATK’s BaseRecalibrator based on known variant sites (NCBI dbSNP). Genome-wide variant calling was performed using GATK’s HaplotypeCaller. To ensure high-confidence SNP calls, specific quality thresholds (FS > 30.0, QD < 2.0) were applied using GATK’s VariantFiltration. High-quality SNPs of the SNP-type variants were selected and filtered solely through GATK’s SelectVariants. We then downloaded haplotype data from 1,241 individuals on SWIM genotype imputation platform [29] and imputed the above genotype files using beagle (v5.4) software [30]. Finally, we filtered SNPs using BCFtools [31] based on two criteria: minimum minor allele frequency (MAF) < 0.1 and imputation quality (DR^2^) > 0.8 for subsequent analysis.

We first used PLINK (v1.90) [32] software to remove the SNPs that do not conform to the Hardy Weinberg equilibrium (–hwe 1e-6), and then screened the SNPs located within 2 kb up/downstream of the immune candidate genes. We performed an association test for each SNP based on a linear model:

$$\boldsymbol Y=\beta_1\boldsymbol X\;+\;\beta_2\boldsymbol T\;+\;\boldsymbol\varepsilon,$$

Where ***Y*** is phenotype value; ***X*** is a vector of genotypes of a variant at the locus tested; *β*_1_ is the effect size of the variant; ***T*** is a vector of age values for the individuals in the study; *β*_2_ is the effect size of age; ***ε*** is a vector of residual errors. Then, we utilized the “–linear” parameter of PLINK to incorporate age as a covariate and performed association analysis on SNPs from 15 PBMC samples of Landrace pigs, using either pathway activity score or gene expression level as the phenotype for each individual.

### Analysis of ATAC-seq and ChIP-seq datasets 

We obtained ChIP-seq and ATAC-seq datasets for eight distinct cell types (CD4αβT, CD4 helper T, CD8 T, gamma delta T, myeloid leukocyte, memory B, naïve B, NK) isolated from porcine PBMCs from the NCBI SRA database (PRJEB51699). Then we used Trim Galore (v0.6.10) [33] and FastQC (v0.11.9, https://github.com/s-andrews/FastQC) software to trim the raw reads and obtain sequence quality reports. The clean reads were mapped to the porcine reference genome (Sscrofa11.1) using Bowtie2 software (v2.4.5) [34] with the default parameters. Mapped reads were further filtered using Samtools (v1.15) [31] view utilities with the parameters “-F 1804 -q 25”. Duplicated alignment reads were removed using the Picard Tools (v2.25.2, https://broadinstitute.github.io/picard/) with the parameter “REMOVE_DUPLICATES = true”. For each ChIP-seq sample, we identified peaks using MACS3 (v3.0.0) with options “-p 0.01” (narrow peak for H3K4me1, H3K4me3, H3K27ac; “–broad –broad-cutoff 0.1”, broad peak for H3K27me3). ATAC-seq peaks were called using MACS3 with the following parameters “–call-summits -p 0.01”. For eight distinct immune cell types, we used intersectBed from bedtools (v2.31.0) [35] with parameter “-u -f 0.5 -r” to define the relatively conserved peaks between cell types used for characterizing PBMCs.

### Hi-C dataset analysis

We utilized a publicly available Hi-C dataset (SRR7585916, SRR7585917) of pig fibroblasts to evaluate the strength of interactions between different regions of the pig genome. Initially, ICE-normalized genome contact matrices and allValidPairs file were generated using HiC-Pro software (http://github.com/nservant/HiC-Pro). Next, the allValidPairs file was converted to “.hic” file format and visualized with the plotgardener (v1.8.3) [36] R package to generate chromatin interaction heatmap.

### TF motif and QTL enrichment analysis

We performed motif enrichment analysis on promoter regions (defined as 1,500 bp upstream and 500 bp downstream of transcription start sites) of the interested gene set using HOMER (v4.11) [37] with the default motif database. Additionally, GWAS SNPs associated with immune-related and nonrelated traits were downloaded from the Pig quantitative trait loci (QTL) Database [38]. We conducted permutation tests (1,000) on SNPs within a 100-kb region upstream and downstream of immune candidate genes using regioneR (v1.34.0) [39] R package to assess the extent of enrichment on the QTL associated with porcine health traits. A score exceeding 0.5 for a specific trait is considered indicative of enrichment.

### Poly(I:C) challenge after the gene interference of primary PBMCs

We employed the previously described method to isolate primary PBMCs from fresh porcine blood, and promptly resuspend them in RPMI-1640 culture medium supplemented with 10% fetal bovine serum (FBS). Subsequently, we inoculated 2–3 × 10^6^ cells into 24-well plates for cultivation. The construction of *IFI44* gene-specific small interfering RNA (siRNA) was completed by Jiangsu Genecefe Biotechnology Co., Ltd. *IFI44*-specific siRNAs were transfected into cells by using Lipofectamine 3000 Transfection Reagent (ThermoFisher Scientific, Beijing, China) following the manufacturer’s recommended procedures. The group treated with transfection reagent and negative control siRNA was used as the control group, while the group treated with transfection reagent and *IFI44*-specific siRNA was used as the treatment group. *IFI44*-specific siRNA sequences are listed as follows:

- Forward: 5′-GCGAAAUAGGACCGCUUAATT-3′
- Reverse: 5′-UUAAGCGGUCCUAUUUCGCTT-3′.

Then, the polyriboinosinic:polyribocytidylic acid [Poly(I:C)] lyophilized powder purchased from Sigma-Aldrich (USA) was dissolved to the working concentration. According to the preliminary experimental results, it was diluted to a concentration of 10 μg/mL for subsequent challenge tests. Following a 24-h transfection period, a 10 µg/mL Poly(I:C) solution was introduced to the cells and thoroughly mixed. The cells were subsequently incubated for an additional 24 h, after which relevant cellular indicators were assessed. An equal amount of cell culture solution was added as the control group for Poly(I:C).

### Real-time PCR and enzyme-linked immunosorbent assay

The total RNA was extracted from the target cell by using the TRIzol reagent (Invitrogen, Carlsbad, CA, USA) in accordance with the manufacturer’s recommendation. Then, RNA was reverse-transcribed using the RT reagent kit (Takara, Shiga, Japan). The mRNA expression level was determined through qRT-PCR by using the SYBR Green I Master mix (Roche, Basel, Swiss) and analyzed on the Roche LightCycler 480 instrument. The GAPDH gene was used as the reference gene of target gene expression. The 2^−ΔΔCt^ method was used to calculate the relative gene expression level. Primers are listed as follows:

- GAPDH-Forward: 5′-GATGACATCAAGAAGGTGGTGA-3′,
- GAPDH-Reverse: 5′-CAGCATCAAAAGTGGAAGAGTG-3′,
- IFI44-Forward: 5′-CCTTTGTGTTCAGTGCCTACTCT-3′,
- IFI44-Reverse: 5′-GAGTGAGCAATACCACCTGTACC-3′.

Then, cytokine expression was assessed in cells transfected for 24 h and treated with Poly(I:C) for 24 h, utilizing enzyme-linked immunosorbent assay (Elisa) kits for porcine IFN-α, IFN-β, IL-6, and TNF-α provided by Shanghai Enzyme-linked Biotechnology Co., Ltd. (Shanghai, China) in accordance with the manufacturer’s recommendation.

### Cell proliferation, apoptosis and necrosis detection

Following a 24-h treatment with poly(I:C), the cells were seeded into 96-well plates at a density of 2 × 10^6^ cells per well, with a volume of 100 µL per well. Afterward, the Apoptosis and Necrosis Detection Kit (Beyotime, Shanghai, China) was used to determine cell apoptosis and necrosis, and the Cell Counting Kit-8 (Beyotime, Shanghai, China) was used to determine cell proliferation. All determination steps were performed following the manufacturer’s instructions. The fluorescence value and absorbance value were determined using the Varioskan LUX (ThermoFisher Scientific). Cells treated with the test kit were photographed by Nikon ECLIPSE Ts2 fluorescence microscope (Nikon, Tokyo, Japan). Apoptotic cells were stained green by YO-PRO-1, and necrosis cells were stained red by PI (Propidium Iodide).

## Results

### Comprehensive transcriptome profile of pig immune tissues

**Fig. 1:**
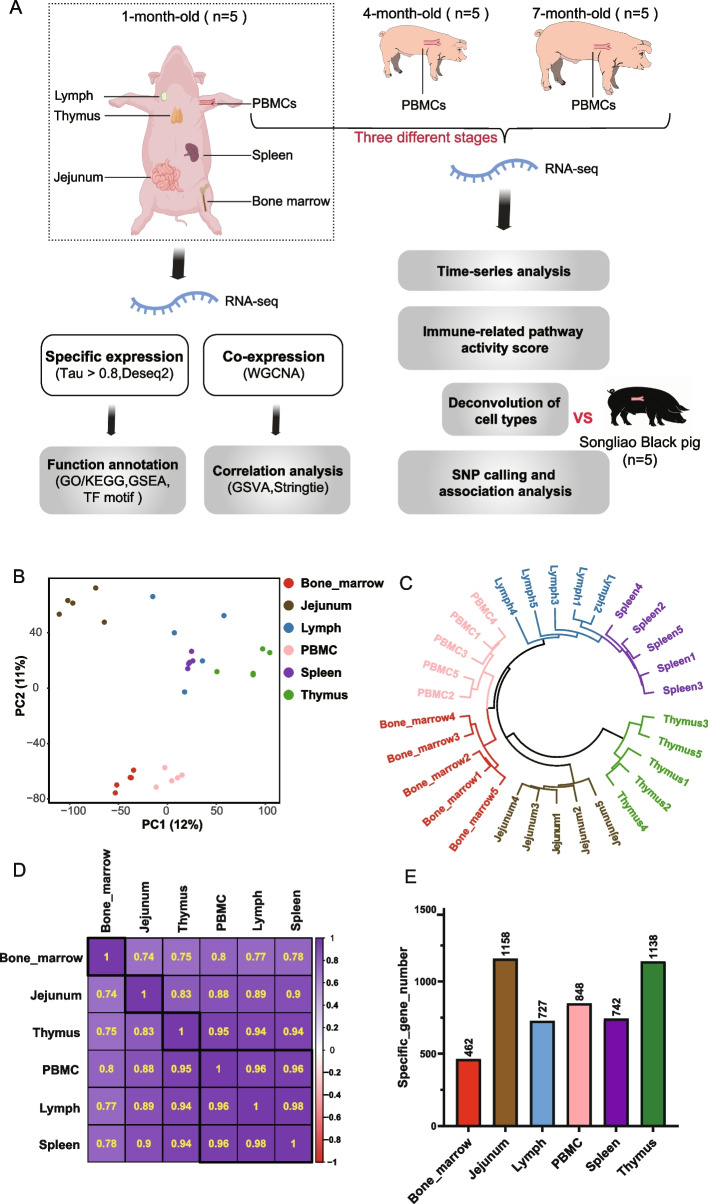
Study design and transcriptome in six pig immune tissues. **A** We collected six kinds of immune tissues from one-month-old Landrace piglets, and analyzed the specific expression and co-expression patterns among the immune tissues to construct a comprehensive immune transcriptome of Landrace pigs; Then we also collected PBMCs of 4-month-old and 7-month-old pigs to analyze the immune dynamics of PBMCs combined with three time points, and collected PBMCs of local breed Songliao Black pig to analyze the immune differences among different breeds. **B** PCA analysis of six immune tissue samples of Landrace piglets based on gene expression levels. **C** Unsupervised clustering of the samples. **D** Pearson correlation (heatmap) of transcriptome profiles across six immune tissues. **E** Histogram of the number of tissue-specific gene expression obtained from six immune tissues (τ > 0.8)

In order to comprehensively understand the expression patterns of immune tissues in pigs, a total of 30 samples (5 replicates per tissue) were collected from various immune organs including bone marrow, jejunum, lymph node, PBMCs, spleen and thymus of Landrace piglets at the age of 1-month. Additionally, to further investigate the immune dynamics of porcine PBMCs in the later stages, samples were also collected from 4- and 7-month-old Landrace pigs. Furthermore, PBMCs were collected from Songliao Black pigs (a local breed) to explore potential immune differences between the two breeds (Fig. 1A). We performed transcriptome sequencing on all 45 samples and obtained more than one billion mapping reads with an average mapping rate of 90.68% (Additional file 1: Table S1). After annotation and quality control, a total of 20,893 genes (TPM ≥ 0.1 in ≥ 20% of samples) obtained for subsequent analysis.

The results of principal component analysis (PCA) revealed a significant separation in the gene expression patterns of 30 samples of Landrace piglets based on immune tissue type (Fig. 1B), indicating distinct tissue-specificity in the gene expression profiles. For the six immune tissues, the unsupervised clustering results showed a close proximity of the spleen to the lymph node (Fig. 1C), which also showed the same trend in the PCA analysis. Then we explored the gene expression similarities among the six immune tissues, and we found that there was a high degree of gene expression similarity between all six immune tissues, and the Pearson correlations between different tissues were all greater than 0.7 (Fig. 1D). Among them, PBMCs, spleen and lymph node have the highest degree of correlation, and the correlation coefficients among the three are all above 0.96. In addition, PBMC has the highest correlation with the other five tissue types (all above 0.8), indicating that PBMCs, as a systemic circulating tissue, may best characterize other immune tissues in terms of immune function. Next, we identified specifically expressed genes in each tissue type based on the tissue-specific index tau value (the higher the τ value, the stronger the tissue specificity, here we selected genes with a τ value greater than 0.8), which provides molecular signatures of different immune tissues (Fig. 1E). The thymus had the fewest tissue-specific genes (462), while jejunum and thymus had the most tissue-specific genes (1,158 and 1,138, respectively).

### Functional differences between pig immune tissues

**Fig. 2:**
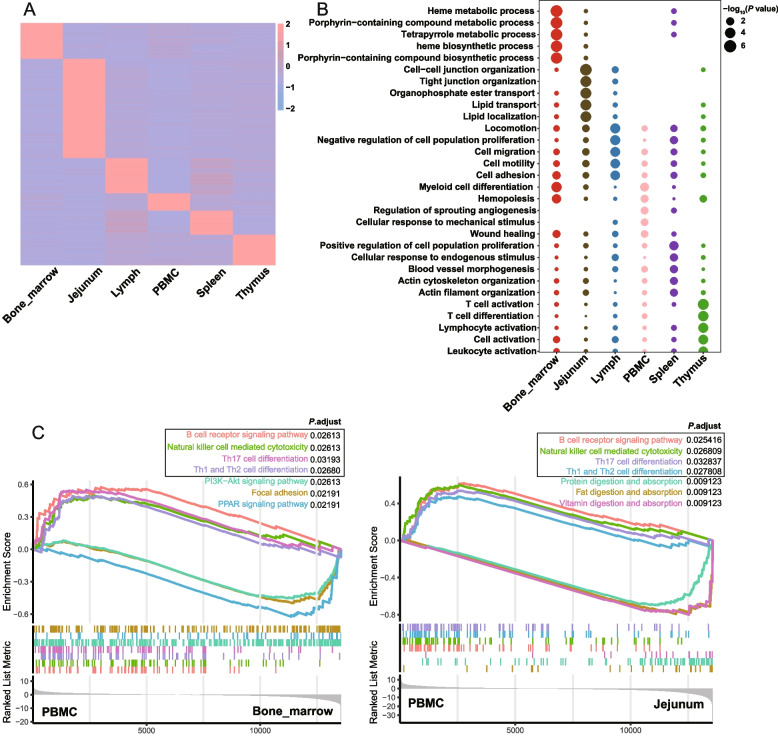
Functional annotation of porcine tissue-specific expression genes. **A** Heatmap showing the expression of tissue-specific expression genes with more than 75% quantile expression in each immune tissues. **B** Dot plot showing the Gene Ontology (GO) pathways of tissue-specific expression genes enrichment in each pig immune tissue (*P* < 0.05). **C** Gene set enrichment analysis (GSEA) showing differences in KEGG pathways (*P*_adjust_ < 0.05) between PBMC and bone marrow (left) and jejunum (right)

In order to explore the functional differences between porcine immune tissues, we further performed GO biological process enrichment analysis on tissue-specific expressed genes with expression levels greater than the 75% quantile (Fig. 2A and B and Additional file 1: Table S2 and S3). The results showed that bone marrow-specific genes were enriched in many pathways related to hemopoiesis (porphyrin-containing compounds and heme metabolism); Jejunum-specific expressed genes were enriched in many pathways related to cell tight junctions, lipid transport, digestion and absorption, and nutrient metabolism; Lymph-specific expressed genes were enriched in pathways related to cell proliferation, migration and adhesion; PBMC-specific expressed genes were enriched in pathways associated with coagulation, immune cell chemotaxis, and immune responses; Spleen-specific expressed genes were enriched in pathways related to hemopoiesis, cell proliferation and activation; Thymus-specific expressed genes were enriched in pathways related to various immune cell activation. Additionally, we performed KEGG enrichment analysis and observed that tissue-specific expression genes were significantly enriched in various disease-related pathways (Additional file 2: Fig. S1). For instance, PBMC-specific expression genes showed enrichment in inflammatory bowel disease, thymus-specific expression genes exhibited enrichment in primary immunodeficiency, and bone marrow-specific expression genes displayed enrichment in transcriptional mis-regulation in cancer. These observations suggested that different immune tissues perform their own duties and play a key role in the body’s immunity and response to environmental stress and disease.

Since PBMC is considered the most representative in terms of expression pattern among various immune tissues, we performed GSEA analyses on PBMC and other immune tissues separately, aiming to explore which immune tissue is more likely to exhibit immune functions. The results demonstrated that PBMCs exhibited significant differential enrichment of immune pathways compared to bone marrow, small intestine, and thymus (Figs. 1D and 2C and Additional file 2: Fig. S2). It can be observed that B cell receptor signaling pathways, natural killer cell mediated cytotoxicity, Th17 cell differentiation and Th1 and Th2 cell differentiation pathways are significantly enriched in PBMCs (Fig. 2C). Although this significant difference in immune pathway enrichment was not observed in the spleen and lymph nodes, it could be attributed to the similarity of immune functions among these three tissues, as analyzed previously. These results lead to the fact that although various immune tissues have corresponding immune functions, PBMC may be the tissue with the stronger characterization ability and immune function.

### Co-expression patterns among pig immune tissues

**Fig. 3:**
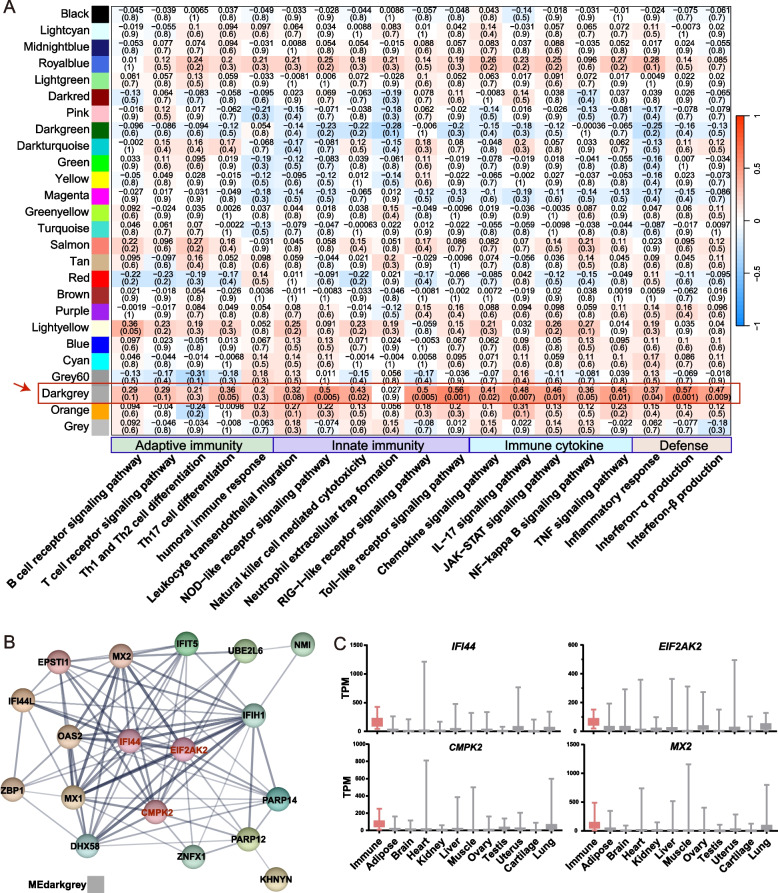
Co-expression module with core immune functions in pig immune tissues. **A** Pearson correlation between modules and immune pathway activity scores calculated based on the eigengene expression. **B** The protein interaction network of genes in the darkgrey module, with a stronger connectivity represented by a redder color. **C** The bar chart showing the TPM expression levels of 4 hub genes *IFI44*, *EIF2AK2*, *CMPK2* and *MX2* in immune tissues and 11 non-immune tissues. The word “Immune” on the *x*-axis represents the average expression level in six types of immune tissues

To search for potential core immune functions among the six immune tissues, we conducted WGCNA to identify co-expression modules in the expression profiles of 30 samples from the six immune tissues. We identified a total of 26 main gene transcriptional modules, with the turquoise module containing the highest number of genes, followed by blue, brown, and yellow (Additional file 2: Fig. S3A). To assess immune differences at the transcriptional level for 30 samples from the six immune tissues, we calculated immune pathway activity scores of each sample, which provides an estimate of the average expression of genes in relevant immune pathways. Then we calculated the module eigengene and evaluate Pearson correlations between each immune pathway and module eigengene. We have identified and categorized 19 immune-related pathways from GO and KEGG databases, encompassing four functional categories: adaptive immunity, innate immunity, immune cytokines, and defense response. The correlation analysis results revealed a significant positive association between the darkgrey module and the immune pathway activity score, suggesting that genes within the darkgrey module may possess core immune functions (Fig. 3A). Among the 19 pathways, 14 pathways have correlation coefficients with the darkgrey module exceeding 0.3. For example, the correlation coefficient with the Toll-like receptor signaling pathway reaches 0.56 (*P* = 0.001) and that with interferon-α production reaches 0.57 (*P* = 0.001).

Subsequently, we calculated the MM scores of 40 genes in darkgrey module to identify hub genes with the highest connectivity (Additional file 1: Table S4), where genes with scores greater than 0.9 were considered as hub genes. Furthermore, we conducted protein network interaction analysis using STRING (https://string-db.org/). The results indicated that *IFI44* (Interferon induced protein 44), *EIF2AK2* (Eukaryotic translation factor 2 alpha kinase 2), *CMPK2* (Cytidine monophosphate kinase 2), and *MX2* (Interferon-Induced GTP-Binding Protein Mx2) as central nodes in the network, all exhibiting MM scores above 0.9 (Fig. 3B). And these four genes are only highly expressed in immune tissues compared with other non-immune tissues (including adipose, brain, heart, kidney, liver, muscle, ovary, testis, uterus, cartilage, lung) (Fig. 3C). Additionally, we performed motif enrichment-analysis on the promoter region of genes in the darkgrey (upstream 1,500 bp, downstream 500 bp), and identified an enrichment of transcription factors belonging to the Forkhead Box family (such as FOXA1, FOXM1, FOXO1), MYB family (such as MYB3R1, MYB3R4), and Zinc Finger Protein family (such as ZNF7) (Additional file 2: Fig. S3B). These transcription factors are known to play crucial roles in cell proliferation, differentiation, and immunity. Here, we identified a set of genes that are co-expressed between immune tissues and have core immune functions.

### Immune function of PBMC was significantly changing with age

**Fig. 4:**
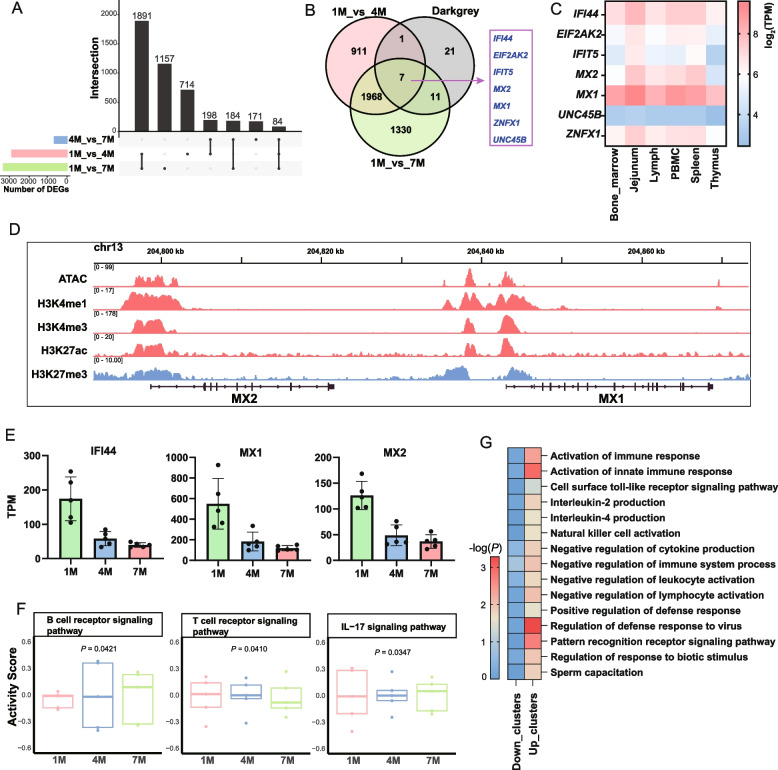
Dynamic changes of PBMC transcriptome at different developmental stages. **A** Upset diagram of porcine PBMC differentially expressed genes between 1-month-old (1M), 4-month-old (4M) and 7-month-old (7M). **B** overlap map of 1M and 4M DEG, 1M and 7M DEG and darkgrey module genes. **C** Heatmap of expression of seven immune candidate genes in six immune tissues. **D** Epigenetic modification peaks (ATAC, H3K4me1, H3K4me3, H3K27ac, H3K27me3) in the promoter regions of *MX1* and *MX2* genes. **E** The gene expression of *IFI44*, *MX1*, and *MX2* in three different developmental stages. **F** Immune pathway activity scores of PBMC samples from different age groups (1M, 4M and 7M)** G** Heatmap of GO enrichment analysis for core genes in up clusters and down clusters

As individuals age, the immune system undergoes a series of adaptive and innate changes that impact susceptibility to diseases and the efficiency of immune responses. Therefore, we further investigated the dynamic changes in transcriptional development of pig PBMC at three stages. We initially utilized DESeq2 to identify the DEGs among 1-month-old (1M), 4-month-old (4M), and 7-month-old (7M) pig PBMCs. Our analysis revealed that the highest number of DEGs was observed between 1M and 7M samples, totaling 3,316 DEGs, while a lower number of DEGs were identified between 4M and 7M, with only 637 DEGs (Fig. 4A). The DEGs were overlapped with the genes identified in the darkgrey module highlighted previously, and 7 immune candidate genes (*IFI44*, *EIF2AK2*, *IFIT5*, *MX2*, *MX1*, *ZNFX1*, *UNC45B*) were discovered (Fig. 4B and E). Among them, genes such as *MX1*, *MX2*, and *IFI44* are highly expressed in six immune tissues, indicating a potential stable immune function (Fig. 4C).

Due to the pivotal role of epigenetic modifications in gene regulation, we conducted further analysis on the distribution of active epigenetic marks (H3K27ac, H3K4me3, H3K4me1) and repressive epigenetic mark (H3K27me3) surrounding these candidate genes. The findings reveal an intriguing pattern, as the majority of promoter regions linked to these candidate genes display a “bivalent chromatin state” [40], characterized by concurrent active epigenetic modifications (H3K27ac, H3K4me1, H3K4me3) and repressive epigenetic modifications (H3K27me3) (Fig. 4D, Additional file 2: Fig. S4AD). The coexistence of inhibiting and activating epigenetic modifications may indicate that these genes are in a plastic state, where they can be further activated or inhibited according to the needs of the cell. This is a regulatory mechanism for responding to environmental stimuli. Subsequently, we further examined the epigenetic modifications of the promoter regions of these candidate genes in eight types of immune cells isolated from PBMCs. We found that *MX1* and *MX2* genes were only modified with H3K27me3 in B cells (memory B, naïve B), myeloid leukocytes and NK cells, but not in T cells (CD4αβT, CD4 helper T, CD8 T, gamma delta T), indicating their primary function in innate immunity (Additional file 2: Fig. S5B); while *IFIT5* gene was only unmodified with H3K27me3 in myeloid leukocytes, and it has been found that overexpression of this gene may be related to myeloid leukemia [41] (Additional file 2: Fig. S5A).

To further explore the patterns of age-related changes in transcriptional levels in PBMCs, we conducted time-series analysis on PBMC samples collected at three distinct stages using Mfuzz R package. A total of 16 gene clusters displaying age-related changes were identified, with clusters 1, 3, 4, 7, 8, and 16 demonstrating a gradual decrease in expression with increasing age. Core genes were extracted from these clusters and defined as down clusters (α > 0.5). Additionally, clusters 9, 10, 11, and 15 exhibited a gradual increase in expression with advancing age while merged together and defined as up clusters (Fig. S6A, Additional file 1: Table S5). The core genes identified in the up and down clusters, along with the DEGs from previous analyses, were overlaped with the genes present in the key module darkgrey. This analysis revealed a significant number of overlapping genes, suggesting their potential involvement in cellular development and differentiation within PBMC (Additional file 2: Fig. S6B and C). Then, we conducted GO term enrichment analysis on the core genes in these gene sets, and found that genes in the up clusters were significantly enriched in immune-related pathways compared with those in the down cluster (such as activation of immune response, cytokine production, negative regulation of immune cell activation, etc.) (Fig. 4G and Additional file 1: Table S6), indicating that with the growth of age, immune function gradually improved and enhanced.

However, it is worth noting that the immune-related pathways show either positive or negative regulation. In order to study the specific changes in immune regulation during development, we determined the immune pathway activity scores of PBMC samples from different age groups (Additional file 1: Table S7). With increasing age, the activity of B cell receptor signaling pathway and B cell activation involved in immune response significantly increased, while the activity of T cell receptor signaling pathway significantly decreased (*P* < 0.05); Th1, Th2 and Th17 cell differentiation pathways showed an increasing trend. In addition, the activity of interleukin-17 signaling pathway and RIG-I-like receptor signaling pathway significantly decreased, while the activity of cellular response to growth factor stimulation pathway significantly increased (Fig. 4F and Fig. S7). These results reflect the changes of innate and adaptive immunity during development, with B cells apparently playing an increasingly crucial role as development progresses.

### The proportion of immune cells in PBMC changed significantly with age

**Fig. 5:**
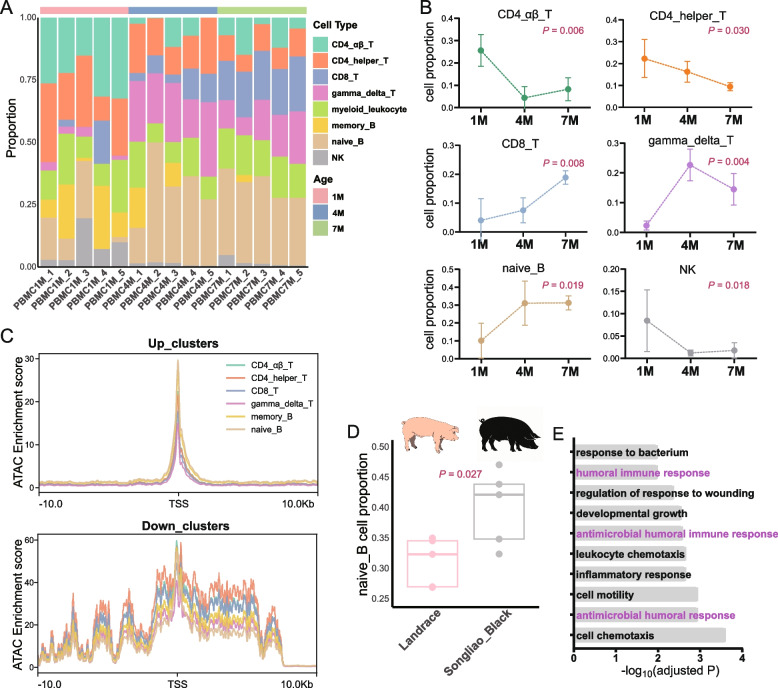
The proportion of porcine immune cells isolated from PBMCs changed with age. **A** Stacked histogram of eight distinct immune cell proportions in 15 PBMC samples. **B** Line chart of cell proportions of CD4αβ T cells, CD4 helper T cells, CD8 T cells, gamma delta T cells, naïve B cells and NK cells changing with age. **C** Distribution of ATAC peaks within ± 10 kb of core gene transcriptional start sites (TSS) in up clusters and down clusters. **D** Box plot of the proportion of naive B cells in PBMC of Landrace pigs and Songliao Black pigs. **E** GO enrichment analysis of DEGs in PBMC of Landrace pigs and Songliao Black pigs. 1M: 1-month-old, 4M: 4-month-old, 7M: 7-month-old

Since the pathway activity of different cell types will change differently with the increase of age, we next investigated the immune cell composition in PBMC at different age stages. To do so, we used transcriptome data from eight immune cells isolated from PBMCs in a public database to deconvolve the proportion of immune cells in each PBMC sample at different age stages using CIBERSORTx. We determined the proportional composition of eight immune cell types in 15 PBMC samples (Fig. 5A). During development, the proportion of CD4αβ T cells and CD4 helper T cells significantly decreased in porcine PBMC, while the proportion of CD8 killer T cells and gamma delta T cells significantly increased. In addition, the proportion of naive B cells significantly increases, while the proportion of NK cells significantly decreases (Fig. 5B). The observed alteration in the T cell to B cell ratio corroborated our previous findings; however, it is noteworthy that there was a significant increase in the proportion of T cells possessing direct pathogenic microorganism-killing abilities, specifically CD8T cells and gamma delta T cells, with gamma delta T cells being an unconventional subset of T cells. This unique population serves as a bridge between innate and adaptive immunity [42]. Additionally, we examined the ATAC peak of the core gene promoter regions in the up and down clusters obtained from the previous analysis. We observed that within the up clusters, there was a heightened chromatin accessibility in B cells within the gene promoter region (Fig. 5C), suggesting a gradual increase in B-cell related gene expression with advancing age. These findings provide further validation for our results.

Due to genetic variations in immunity among different breeds, we also collected PBMC samples from local breed Songliao Black pigs and compared them with commercial breed Landrace pigs. The findings revealed a significantly higher proportion of naive B cells in Songliao Black pigs compared to Landrace pigs (Fig. 5D). Furthermore, the DEGs between these two breeds were significantly enriched in terms of humoral immune response, antimicrobial humoral immune response, antimicrobial humoral response and inflammatory response (Fig. 5E), which suggests that local breed Songliao Black pigs may possess stronger disease resistance [43] due to enhanced humoral immunity, wherein B cells play a pivotal role.

### Immune key genes were enriched with QTL and genetic variation of immune traits

**Fig. 6:**
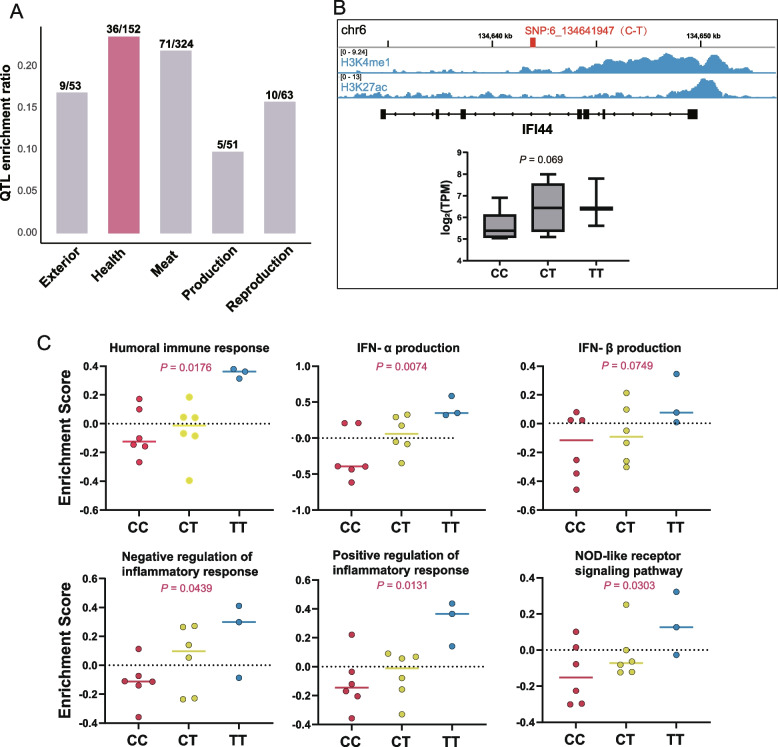
The potential regulatory variants of immune core genes are enriched in QTLs of porcine complex traits and significantly correlated with immune pathway activity score. **A** Bar chart of the enrichment ratio of SNPs within a ±100 kb region surrounding the immune core genes with the QTL data of five pig phenotypes. The numerator represents the number of QTLs significantly enriched with SNPs, and the denominator represents the total number of QTLs.** B** Potential regulatory SNP (6_134641947) in *IFI44* gene body region. The position of the SNP has peaks of H3K4me1 and H3K27ac. Individuals with the T genotype show higher gene expression (*P* = 0.069). **C** The potential regulatory SNP (6_134641947) of *IFI44* was significantly associated with multiple immune pathway activity score, and individuals with T genotype show higher activity scores

In order to further investigate the potential relationship between the functions of previously identified immune core genes and complex traits, particularly those related to health, we identified SNPs from PBMC transcriptome data. The SNPs within a ±100 kb region surrounding the immune core genes in the darkgrey module were searched for co-location enrichment with the QTL datasets of five pig traits obtained from the pig QTL database using permutation tests (1,000 times) (https://www.animalgenome.org/cgi-bin/QTLdb/SS/index). The results showed that the SNPs enriched to the highest proportion of health traits, and are also in many other traits, such as meat and reproduction, suggesting that SNPs are often pleiotropic and may affect both animal health and production traits (Fig. 6A).

Next, we used the PBMC transcriptome data to extracted SNPs mapping less than 10 kb away from immune core genes and then used a linear model to assess the association between their genotypic classes and individual immune pathway activity scores. We found that SNPs with *P* value less than 0.05 obtained by linear analysis were significantly enriched in promoter flanks and enhancer regions (Additional file 1: Table S8, Additional file 2: Fig. S8). Meanwhile, we found many important SNP sites that may be related to immunity. For example, we identified an intronic SNP within the genomic region of the *IFI44* gene: 6_134641947(C-T), which is situated at a location exhibiting both H3K4me1 and H3K27ac peaks, suggesting its potential presence in an enhancer region and possibly serving as an eQTL for *IFI44* (*P* = 0.069) (Fig. 6B). The T genotype individuals in this SNP exhibited significantly elevated immune pathway activity scores, including humoral immune response, IFN-α production, IFN-β production, negative regulation of immune response, positive regulation of immune response, and NOD-like receptor signaling pathway (Fig. 6C). This suggests that their immune function was markedly superior to that of individuals with the C genotype.

Additionally, we also identified two SNPs around *MX1* that are significantly associated with immunity, one of which (SNP:13_204856970(C-T) is located in the gene body region of *MX1*, and the individuals with the C genotype of this SNP show higher gene expression and significantly higher immune pathway activity score (such as IFN-α production, IFN-β production, positive regulation of immune response and TNF signaling pathway) (Additional file 2: Fig. S9AC). Another SNP: 13_204878568(C-T) of *MX1* is located downstream of the gene, and it is noteworthy that this specific SNP location exhibits distinct H3K4me1 and ATAC peaks (Additional file 2: Fig. S9A). Although this SNP did not exhibit a significant association with the immune pathway activity score (such as inflammatory response (*P* = 0.0695), IFN-α production (*P* = 0.1206), IFN-β production (*P* = 0.0672), positive regulation of immune response (*P* = 0.0538)), it may be related to insufficient individuals leads to uneven distribution of individual genotypes (CC genotype:14, CT genotype:1). We also have an example of a SNP downstream of *EIF2AK2* gene: 3_103105200(T-C), in which individuals with the T genotype show higher gene expression and higher immune pathway activity score (Additional file 2: Fig. S10AC). Finally, to further validate the potential regulatory role of SNPs on their target genes, we obtained and analyzed porcine fibroblast Hi-C data from public. Surprisingly, both SNPs surrounding *MX1* exhibited strong chromatin interactions with the promoter region of *MX1* (Additional file 2: Fig. S9A). Moreover, two SNPS surrounding the *IFIT5* gene were also found to have strong chromatin interactions with the gene promoter region (Additional file 2: Fig. S11). Overall, these observations suggest that the core immune genes obtained by analyzing the comprehensive six porcine immune tissue are closely related to the complex traits of pigs, especially immunity, and the identification of the target causal variants behind them can accelerate the advancement of genetic breeding for disease resistance.

### *IFI44* gene affects the apoptosis and necrosis of porcine primary PBMCs induced by Poly(I:C) challenge

**Fig. 7:**
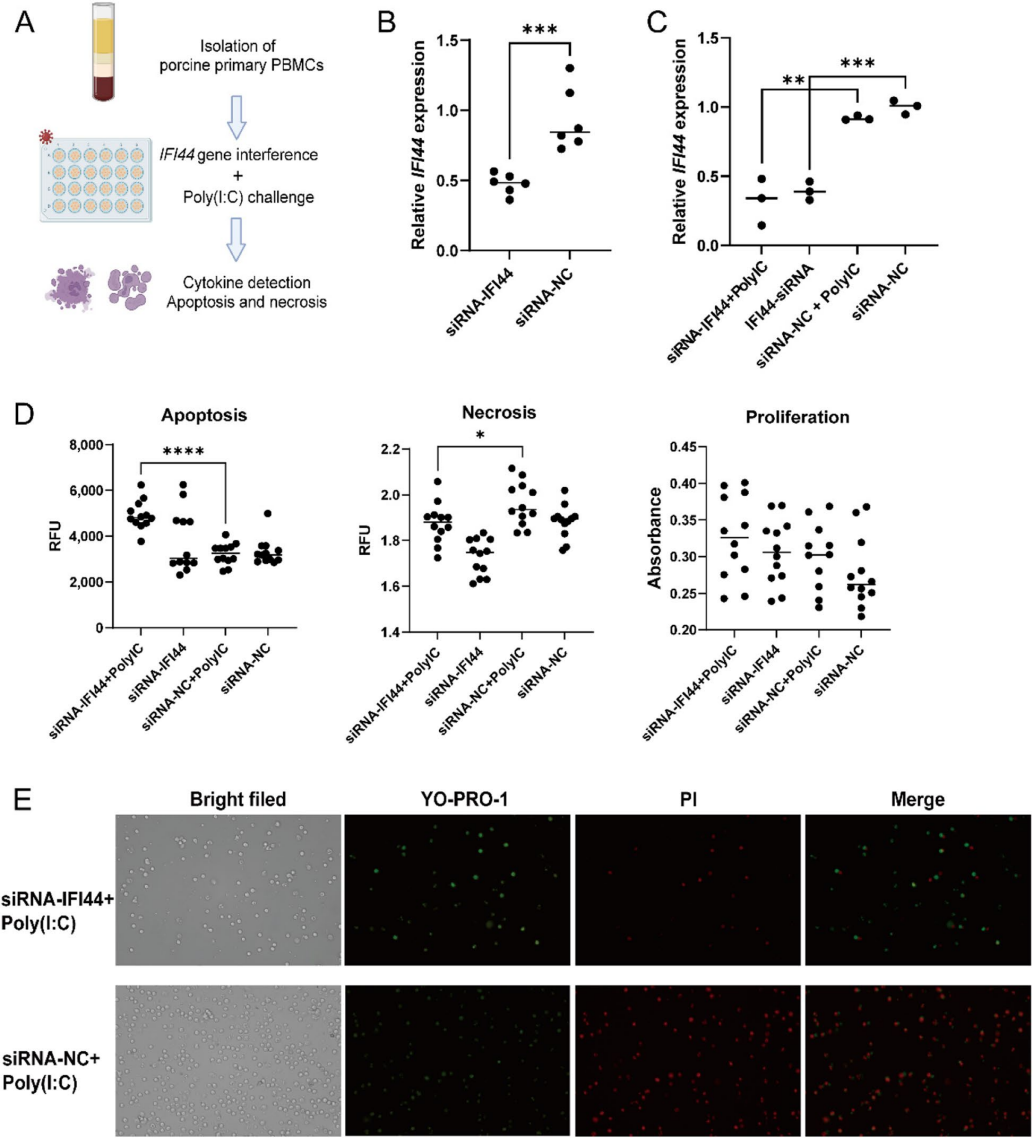
Involvement of *IFI44* gene in the regulation of PBMC apoptosis and proliferation induced by Poly(I:C) challenge. **A** Workflow of *IFI44* gene function validation. **B** The relative expression of *IFI44* gene after 24 h transfection with small interfering RNA. **C** The relative expression level of *IFI44* gene stimulated by Poly(I:C) for 24 h. **D** The interference with the *IFI44* gene regulated the cell apoptosis, necrosis and cell proliferation induced by Poly(I:C). **E** Cell apoptosis and necrosis was assessed with fluorescence microscopy. Apoptotic cells were stained green by YO-PRO-1, and necrosis cells were stained red by PI (Propidium Iodide). siRNA-IFI44: IFI44 gene interference group. siRNA-NC: negative control. siRNA-IFI44 + Poly IC: After interfering *IFI44* gene for 24 h, the treatment group was challenged with Poly(I:C). siRNA-NC + Poly IC: The negative control group was challenged with Poly(I:C) after 24 h

Considering the significant association of the SNP site within the *IFI44* gene with the interferon-producing immune pathway in antiviral response, *IFI44* gene was chosen for the experiment of gene interference and Poly(I:C) challenge to confirm its role in regulating cellular immune response (Fig. 7A). Initially, the interference with the *IFI44* gene for a duration of 24 h resulted in a significant reduction in the expression of the *IFI44* gene (Fig. 7B), and this knockdown effect persisted for up to 24 h following Poly(I:C) challenge (Fig. 7C). Then we studied the effect of interfering with the *IFI44* gene on cytokine production, and the results showed that knocking down *IFI44* gene would lead to a trend of increased expression of IFN-α and IFN-β, a significant decrease in TNF-α, and a reduction in IL-6 (Additional file 2: Fig. S12A and B). It is worth noting that the interference with the *IFI44* gene remarkably reduced the cell necrosis induced by Poly(I:C) challenge, while significantly increased the cell apoptosis (Fig. 7D). Furthermore, there was a discernible inclination towards increased cell proliferation after knocking down *IFI44* gene (Fig. 7D). The results of fluorescence staining also verified the function of knocking down *IFI44* gene on cell apoptosis and cell necrosis induced by Poly(I:C) challenge (Fig. 7E).

## Discussion

The gene expression of different tissues is closely associated with their specific complex traits [44]. For instance, Gondret et al. identified the biological pathways and genes related to pig feed efficiency through transcriptome multi-tissue analysis on tissues (liver, muscle, adipose) closely related to metabolism [45]. The pigGTEx project has conducted studies on 34 tissues of pigs [10]. However, due to the large amount of data and complex statistical analysis, the regulatory mechanisms of immune-specific traits still not deeply understood. Here, we conducted a systematic study on six representative porcine immune tissues, aiming to explore the core transcriptional programs related to immune phenotypes. Leveraging transcriptome data obtained from PBMCs at three developmental stages, alongside multi-omics data from 8 distinct types of immune cells isolated from PBMCs, we further elucidated the dynamic changes in pig immune development process.

The immune system is an interactive network of immune tissues, immune cells and cytokines, which plays a crucial role in the host defense against pathogens and diseases [46]. The immune organs of pigs are the tissue structures that carry out the immune function of the pig’s body, including primary immune organs (bone marrow, thymus) and secondary immune organs (blood, spleen, lymph node) [47, 48]. The two types of organs are closely connected, with the primary immune organs being the sites where B and T lymphocytes differentiate and mature, and the secondary immune organs receiving lymphocytes transported from the primary immune organs, serving as the main sites for carrying out immune responses [49]. In addition, the intestine, as the largest immune organ in the body, has gut-associated lymphoid tissue (especially in small intestine) and a large number of immune cells. Previous studies have shown that gene expression is clearly tissue-specific [50], even between different species [51]. We found that unsupervised gene expression clustering was essentially able to differentiate between these six types of tissues, despite all belonging to immune tissues. However, there is also strong similarity between immune tissues, with the transcriptional correlation between PBMC and the other five tissues all reaching above 0.8, and the similarity between PBMC and spleen, lymph nodes even reaching as high as 0.96. These results suggest that gene expression similarities between immune tissues stem from their functional similarity. Among them, blood as a circulating tissue, not only provides nutrients for other organs, but also transports immune cells produced by various organs [52–54], which is considered to be the most representative immune tissue. The dynamics and migration of immune cells in PBMCs isolated from blood play an important role in the body’s immune regulation [55, 56], and in our results, PBMCs indeed showed a stronger enrichment of immune related KEGG pathways compared to other five tissues. In addition, this study identified a core co-expression module closely related to immune function by analyzing the expression patterns of six immune tissues. The hub genes in this module, such as *IFI44*, *EIF2AK2*, *CMPK2*, and *MX2*, are only highly expressed in immune tissues. The genes in this module are closely related to innate immunity, adaptive immunity and cytokine production. We regard them as the core immune genes, which may be linked to the individual’s disease resistance. For example, it was found that the *IFI44* gene can inhibit the replication of respiratory syncytial virus [57], and the *EIF2AK2* gene is associated with a variety of human diseases [58–60]. In addition, *MX1* and *MX2* genes, as sticky antiviral proteins, have also been found to have an important relationship with antiviral function [61, 62].

From birth to adulthood, the immune system undergoes a series of complex changes, including the development and functional maturation of organs, such as the development of T cells in the bone marrow and thymus, and the maturation of B cells [63–65]. Studying the development of an individual’s immune system is key to gaining a deeper understanding of the mechanisms that perform functional immune function. Blood, as a circulating tissue, contains various immune cells, particularly PBMC. Exploring the dynamic changes in their development is indicative to help us better understand the changes in the body’s immune response. We found that the transcriptome of PBMC undergoes drastic changes in the early stages of development and then gradually stabilizes, which is consistent with previous studies [66]. Many of the genes associated with development overlapped with genes in core immune modules identified from six immune tissues. Interestingly, there is a “bivalent chromatin state” in the promoter region of these genes, which was first identified in stem cells [67]. Generally speaking, during development, mammalian cells display a paradoxical chromatin state: histone modifications associated with gene activation (H3K4me3) and with gene repression (H3K27me3) co-occur at promoters of developmental genes. This bivalent chromatin modification state is thought to poise important regulatory genes for expression or repression during cell-lineage specification [68]. In this research, the observed chromatin state was more likely a regulatory mechanism of the immune system, where core immune genes are modulated to respond more rapidly to external stimuli. In turn, looking for this chromatin pattern helps anchor key immune genes or regulatory variants in the genome on the basis of immunophenotypic related analytical data, which may be a novel way for us to identify disease resistance genes.

In addition, during development, the genes in up clusters were significantly related to immune pathways, indicating that the function of the immune system is gradually maturing. By analyzing immune pathway scores and the proportion of immune cells at three different periods of PBMC, we found that newborns are more dependent on the innate immune system, while adaptive immunity matures with age, such as the increase in the proportion of B cells [69]. We also found that the proportion of B cells in PBMCs of Songliao Black pigs was significantly higher than that of Landrace pigs, suggesting that the difference in immunity between breeds may be due to the strength of humoral immunity. Afterwards, we observed that SNPs within a ± 100 kb region surrounding the core immune genes were significantly enriched for QTLs related to porcine complex traits, among which the proportion of enriched healthy traits was the highest, indicating that the core immune genes may regulate individual phenotypes. We also found many potential regulatory causal variations around core immune genes, which were significantly related to the production of individual cytokines and the strength of the body’s immune response. Therefore, these results indicate that comprehensive analysis of the transcriptome of all representative immune tissues of pigs can reveal core genes related to immune phenotypes, which will facilitate the discovery of relevant causal variants.

The pig farming industry has been persistently affected by viral diseases [70], with type I interferon(IFN) serving as a crucial cytokine in the antiviral response within the host, which is able to activate a second round of autocrine and paracrine signals, that allows infected and surrounding cells to activate anti-viral programs mediated by Interferon Stimulated Genes [71]. Previous studies have reported that the *IFI44* gene plays a crucial role in the innate immune response [72], and is associated with the antiviral mechanisms [57]. Our results elucidated that the silencing of the *IFI44* gene led to an enhanced secretion of type I interferon cytokines, particularly IFN-α and IFN-β, by PBMC. These findings suggest that *IFI44* functions as a negative regulator of type I interferon, which aligns with previous reports [73]. Meanwhile, after interfering with the *IFI44* gene, the secretion of pro-inflammatory cytokines TNF-α was significantly reduced, and IL-6 levels were also decreased, suggesting that *IFI44* may promote inflammatory responses. The absence of *IFI44* may reduce excessive inflammatory responses, which may have a protective role in pathological conditions such as viral infections. Notably, the interference of the *IFI44* gene markedly diminished cell necrosis induced by Poly(I:C), while simultaneously enhancing apoptotic processes. Necrosis is a passive form of cell death that can trigger a strong inflammatory response [74], and pathogens such as viruses can significantly cause cell necrosis [75]. Our results show that inhibition of the *IFI44* gene mitigated necrosis induced by Poly(I:C), thereby alleviating the excessive inflammatory response and tissue damage associated with viral infection. Apoptosis is a programmed cell death, and apoptotic cells can be quickly engulfed by macrophages to prevent the release of intracellular components [76]. After disrupting the *IFI44* gene, the cell apoptosis induced by Poly(I:C) was significantly increased, indicating that the body may have initiated an apoptosis program to try to eliminate damaged cells stimulated by Poly(I:C) in order to minimize the inflammation area and prevent spread. Overall, the *IFI44* gene functions as a negative regulator and is crucial in modulating the antiviral response.

## Conclusion

Collectively, our study systematically identified the core immune genes and potential causal variations in pig immune tissues, among which *IFI44* has been experimentally validated as a virus susceptibility gene. Furthermore, this investigation revealed that PBMC is the most representative immune tissue and easily accessible for collection in practical breeding efforts. The utilization of its immune pathway activity score enables us to quantitatively assess the immunophenotype and establish a theoretical foundation for disease resistance breeding in pigs.

## Supplementary Information

Additional file 1: Table S1. Sequencing and mapping statistics of RNA-seq data. Table S2. Tissue-specific expressed genes with expression levels greater than the 75% quantile. Table S3. Significantly enriched GO terms for tissue-specific expressed genes with expression levels greater than the 75% quantile. Table S4. Darkgrey module genes with module membership score. Table S5. The core genes in the 16 gene sets obtained from time series analysis (α > 0.5). Table S6. Significantly enriched GO terms for core genes in up clusters and down clusters. Table S7. Immune-related pathway activity score of 15 PBMC samples. Table S8. SNPs with *P*-value less than 0.05 correlated with individual immune pathway activity scores.

Additional file 2: Fig. S1. Significantly enriched KEGG pathways for tissue-specific expressed genes. Fig. S2. GSEA analyses on PBMC and other immune tissues (Thymus, Lymph, Spleen). Fig. S3. A Major gene transcription modules were identified through WGCNA analysis. B Motif enrichment analysis on the promoter region of genes in the darkgrey module (upstream 1,500 bp, downstream 500 bp). Fig. S4. The “bivalent chromatin state” observed in the promoter regions of immune candidate genes. Fig. S5. Epigenetic modification of H3K27me3 peaks around immune candidate genes MX1, MX2, and IFIT5 in eight immune cells. Fig. S6. A 16 gene clusters displaying age-related changes. B and C The core genes identified in both the upregulated and downregulated clusters, together with the DEGs from prior analyses, were compared against the genes present in the key module designated as dark grey. Fig. S7. Immune pathway activity scores of PBMC samples from different age groups (1M, 4M and 7M). Fig. S8. Enrichment of SNPs around immune core genes in 15 chromatin states. Fig. S9. Two SNPs around MX1 identified that are significantly associated with immunity. A Chromatin interaction and four epigenetic modifications around the *MX1* gene. B and C SNP:13_204856970(C-T)) is located in the gene body region of MX1 , and the individuals with the C genotype of this SNP show higher gene expression and significantly higher immune pathway activity score. Fig. S10. One SNP (3_103105200(T-C) around EIF2AK2 identified that was significantly associated with immunity. A Peak map of two epigenetic modifications around this SNP. B Gene expression of different genotypes of this SNP. C Immune pathway activity scores of different genotypes of this SNP. Fig. S11. Two SNPs around IFIT5 identified that are significantly associated with immunity. A Chromatin interaction and four epigenetic modifications around the IFIT5 gene. B Gene expression and immune pathway activity scores of different genotypes of the SNP:14_101270261(C-T). C Immune pathway activity scores of different genotypes of the SNP:14_101264262(T-C). Fig. S12. The effect of interfering with the IFI44 gene on cytokine production. A The concentrations of 4 cytokines were detected after 24 hours of interference with IFI44 gene. B After 24 h of interference with IFI44 gene, Poly(I:C) was used for stimulation, and the concentrations of 4 cytokines were detected after another 24 h.

## Data Availability

Raw sequencing data for the eight samples analyzed in this study have been uploaded to the Sequence Read Archive (SRA) in National Center for Biotechnology Information (NCBI), and are available under accession number: PRJNA562774 or (https://www.ncbi.nlm.nih.gov/bioproject/PRJNA562774). Data generated during analysis are included in the manuscript as supplementary files.

## Abbreviations

DEG: Differentially expressed gene
FBS: Fetal bovine serum
GO: Gene Ontology
GSEA: Gene set enrichment analysis
KEGG: Kyoto Encyclopedia of Genes and Genomes
PBMC: Peripheral blood mononuclear cell
PCA: Principal component analysis
Poly(I:C): Polyriboinosinic:polyribocytidylic acid
PRRSV: Porcine reproductive and respiratory syndrome virus
siRNA: Small interfering RNA
SNP: Single nucleotide polymorphism
TPM: Transcripts per kilobase per million mapped reads
TSS: Transcriptional start sites
WGCNA: Weighted correlation network analysis
1M: 1-Month-old
4M: 4-Month-old
7M: 7-Month-old
