# Supplementary material for: The comprehensive transcriptomic atlas of porcine immune tissues and the peripheral blood mononuclear cell (PBMC) immune dynamics reveal core immune genes
Source: J Anim Sci Biotechnol. 2025 May 19;16:69. doi: 10.1186/s40104-025-01184-y (PMC12087129; doi:10.1186/s40104-025-01184-y)
Supplement: Supplementary file 2 — Additional file 2: Fig. S1. Significantly enriched KEGG pathways for tissue-specific expressed genes. Fig. S2. GSEA analyses on PBMC and other immune tissues (Thymus, Lymph, Spleen). Fig. S3. A Major gene transcription modules were identified through WGCNA analysis. B Motif enrichment analysis on the promoter region of genes in the darkgrey module (upstream 1,500 bp, downstream 500 bp). Fig. S4. The “bivalent chromatin state” observed in the promoter regions of immune candidate genes. Fig. S5. Epigenetic modification of H3K27me3 peaks around immune candidate genes MX1, MX2, and IFIT5 in eight immune cells. Fig. S6. A 16 gene clusters displaying age-related changes. B and C The core genes identified in both the upregulated and downregulated clusters, together with the DEGs from prior analyses, were compared against the genes present in the key module designated as dark grey. Fig. S7. Immune pathway activity scores of PBMC samples from different age groups (1M, 4M and 7M). Fig. S8. Enrichment of SNPs around immune core genes in 15 chromatin states. Fig. S9. Two SNPs around MX1 identified that are significantly associated with immunity. A Chromatin interaction and four epigenetic modifications around the MX1 gene. B and C SNP:13_204856970(C-T)) is located in the gene body region of MX1 , and the individuals with the C genotype of this SNP show higher gene expression and significantly higher immune pathway activity score. Fig. S10. One SNP (3_103105200(T-C) around EIF2AK2 identified that was significantly associated with immunity. A Peak map of two epigenetic modifications around this SNP. B Gene expression of different genotypes of this SNP. C Immune pathway activity scores of different genotypes of this SNP. Fig. S11. Two SNPs around IFIT5 identified that are significantly associated with immunity. A Chromatin interaction and four epigenetic modifications around the IFIT5 gene. B Gene expression and immune pathway activity scores of different genotypes of the SNP:14_1 [file 40104_2025_1184_MOESM2_ESM.docx]

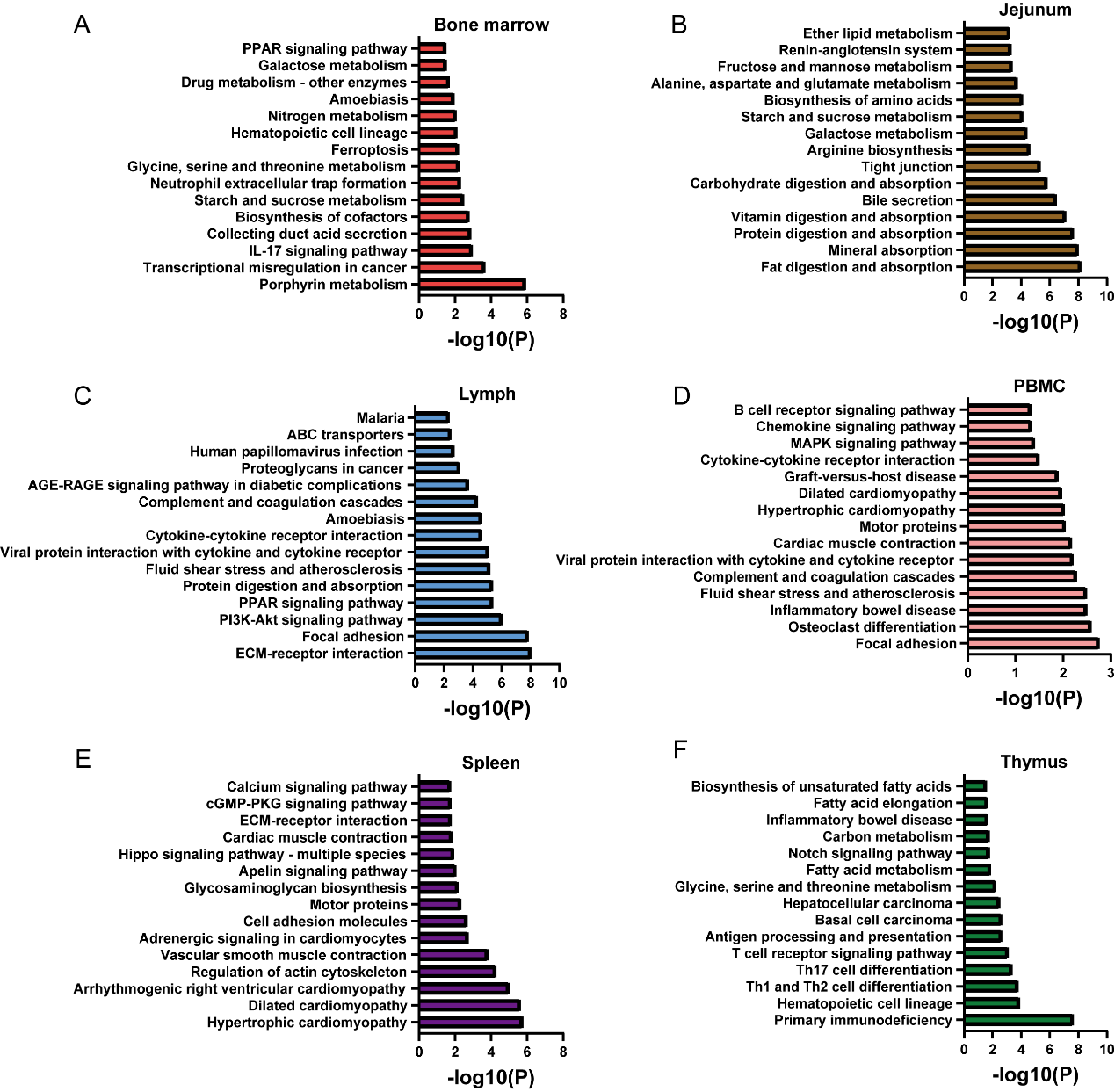


**Fig. S1** Significantly enriched KEGG pathways for tissue-specific expressed genes


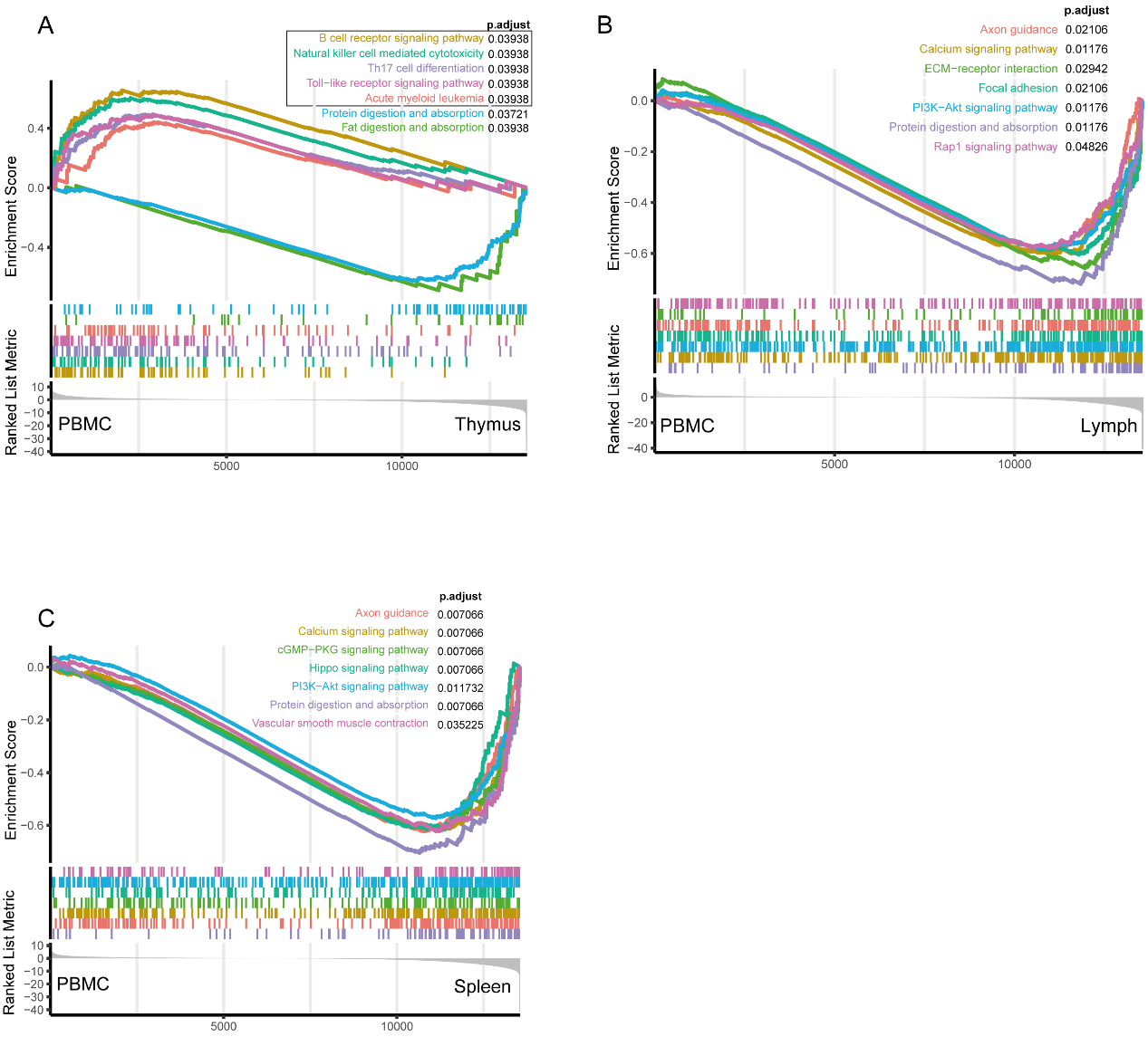


**Fig. S2** GSEA analyses on PBMC and other immune tissues (Thymus, Lymph, Spleen)


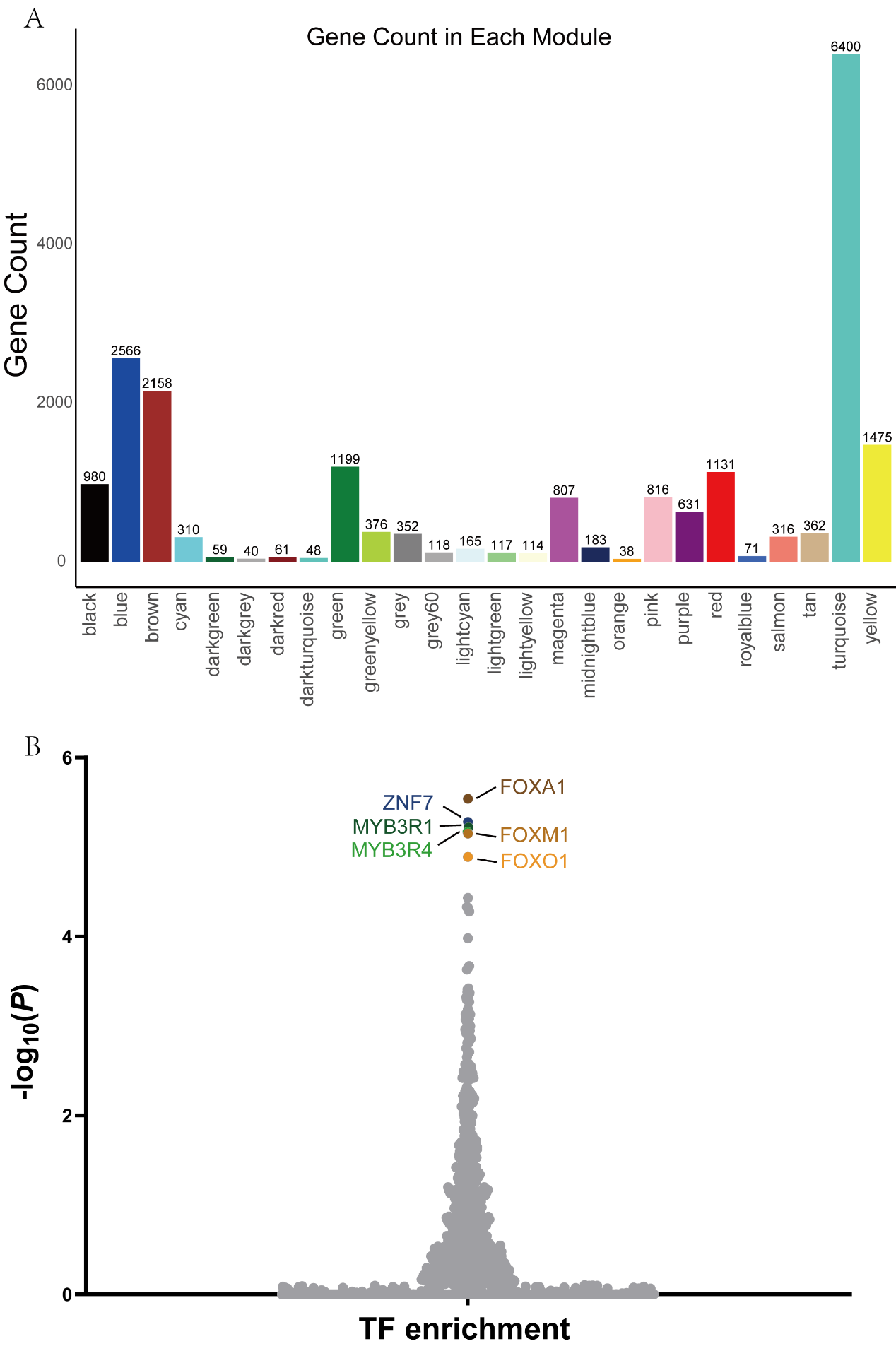


**Fig. S3** **A** Major gene transcription modules were identified through WGCNA analysis. **B** Motif enrichment analysis on the promoter region of genes in the darkgrey module (upstream 1,500 bp, downstream 500 bp)


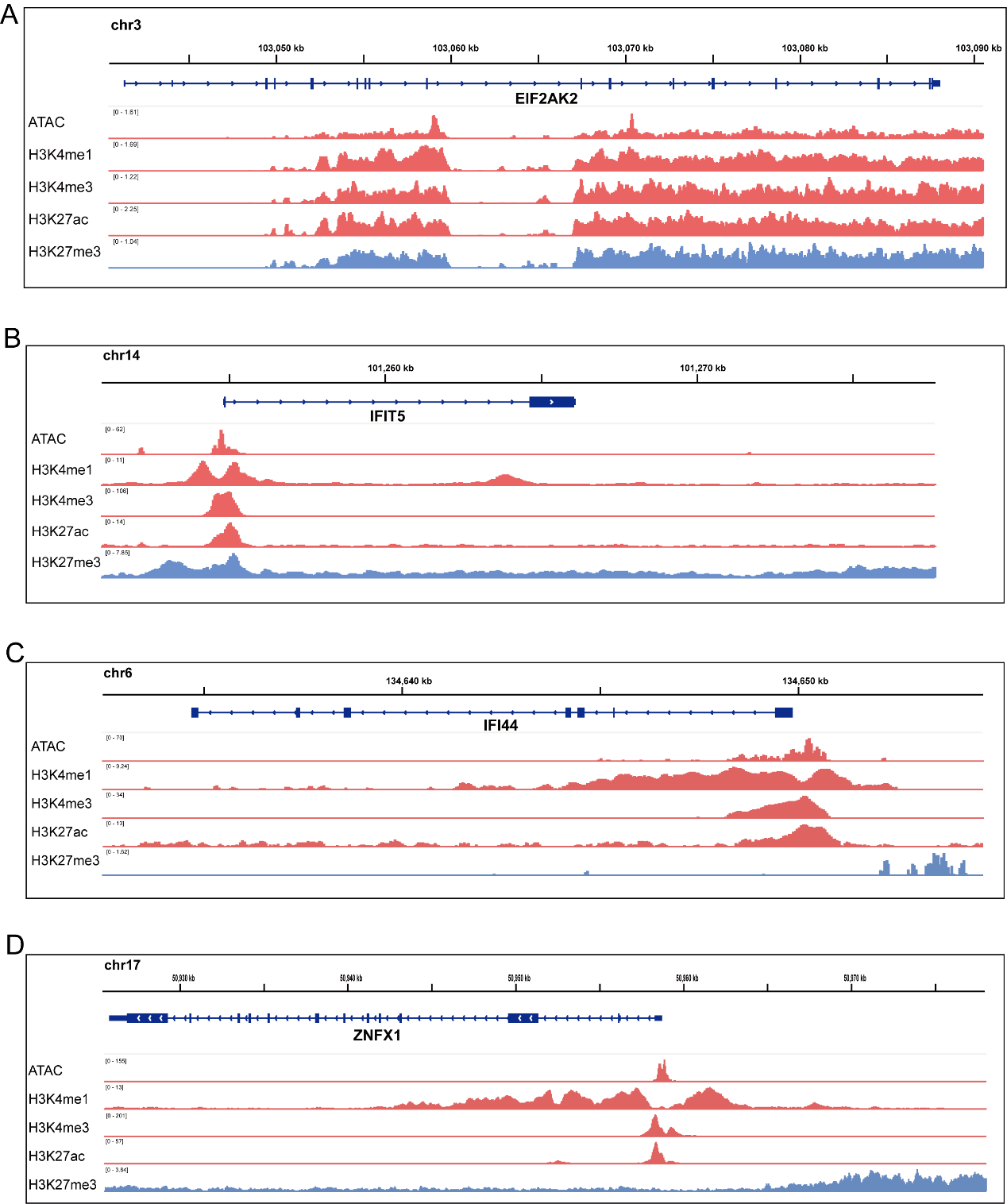


**Fig. S4** The “bivalent chromatin state” observed in the promoter regions of immune candidate genes


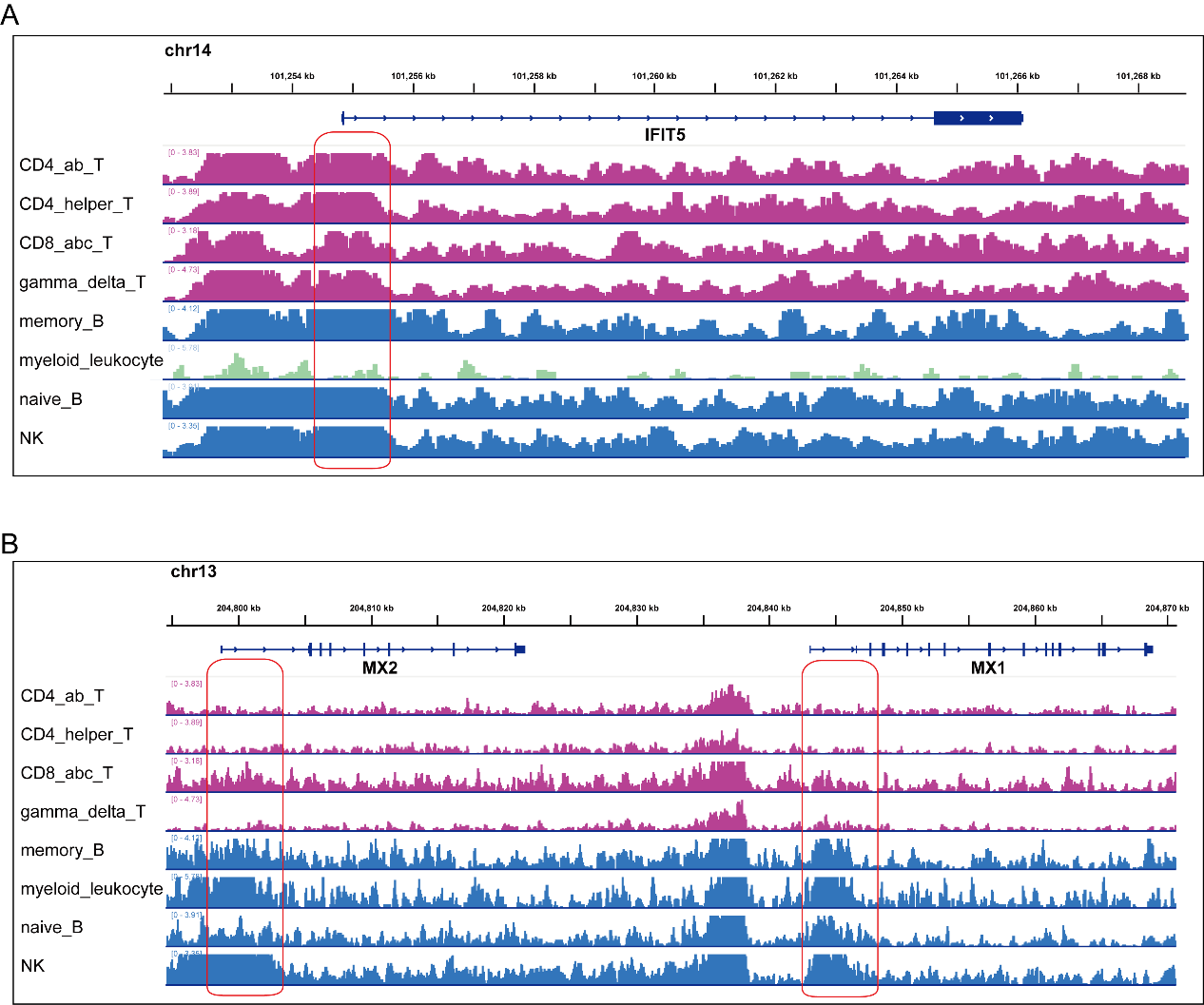


**Fig. S5** Epigenetic modification of H3K27me3 peaks around immune candidate genes in eight immune cells
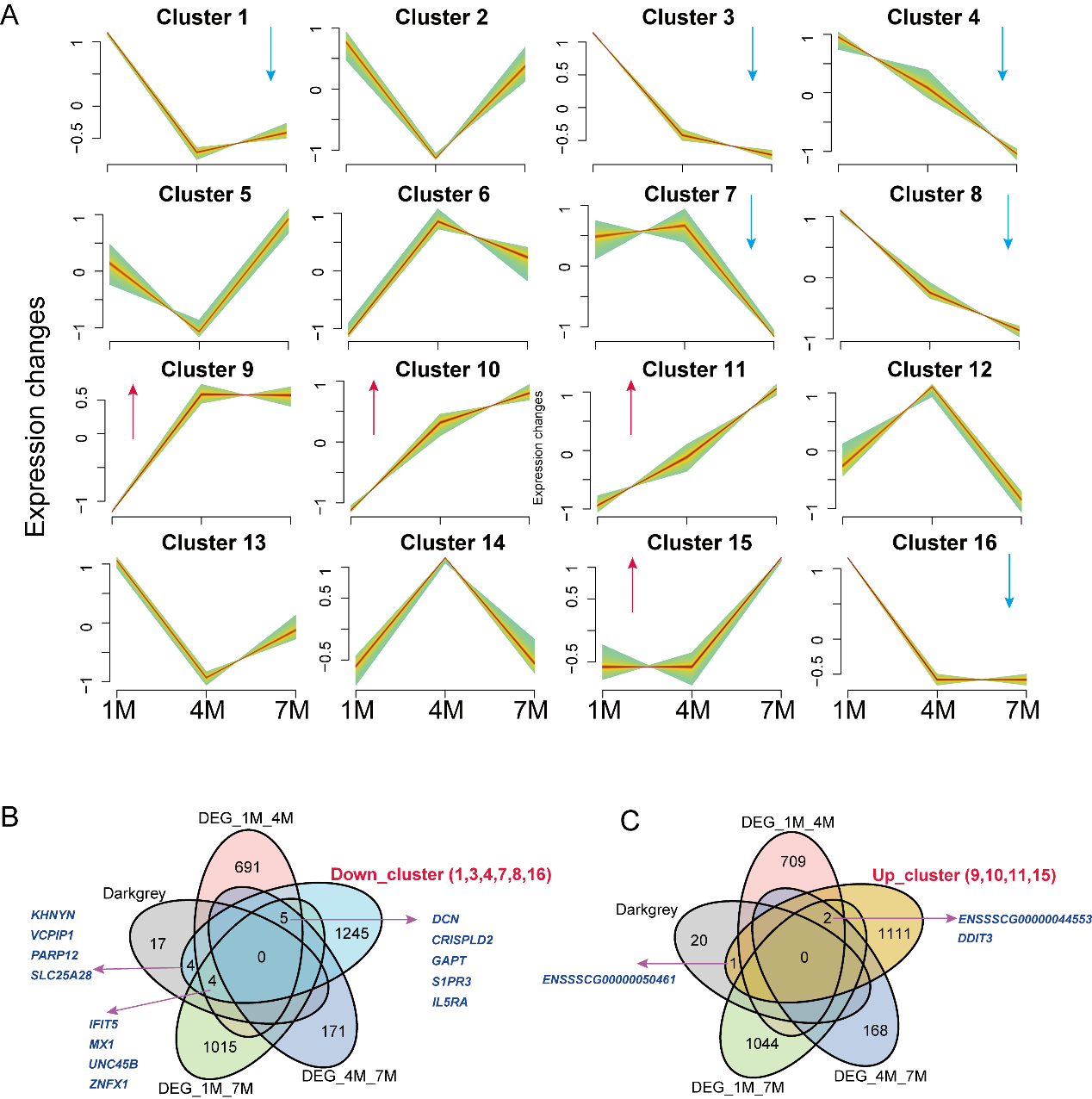


**Fig. S6** **A** 16 gene clusters displaying age-related changes **B** and **C** The core genes identified in both the upregulated and downregulated clusters, together with the DEGs from prior analyses, were compared against the genes present in the key module designated as dark grey

**
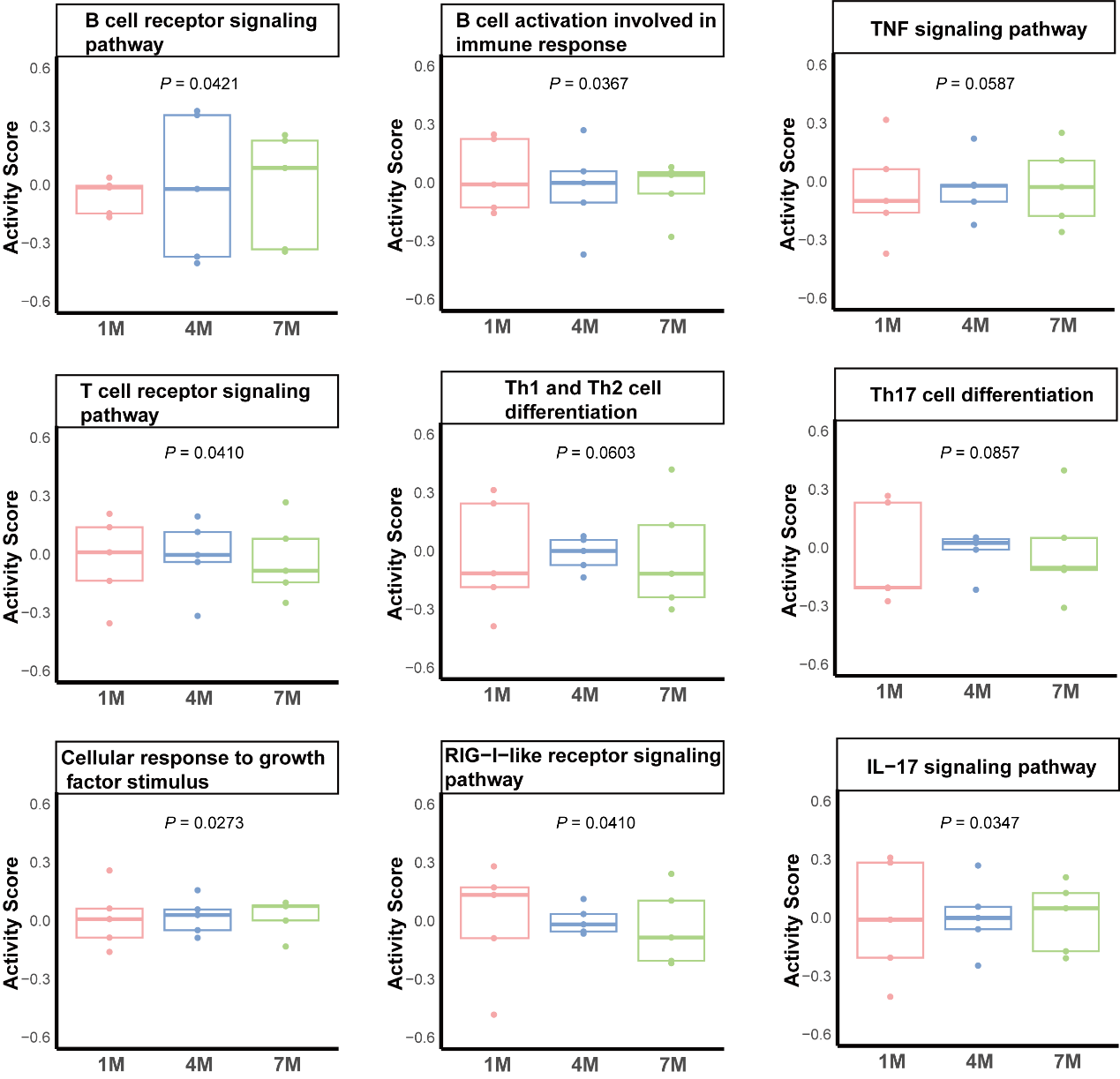
**

**Fig. S7** Immune pathway activity scores of PBMC samples from different age groups (1M, 4M and 7M)


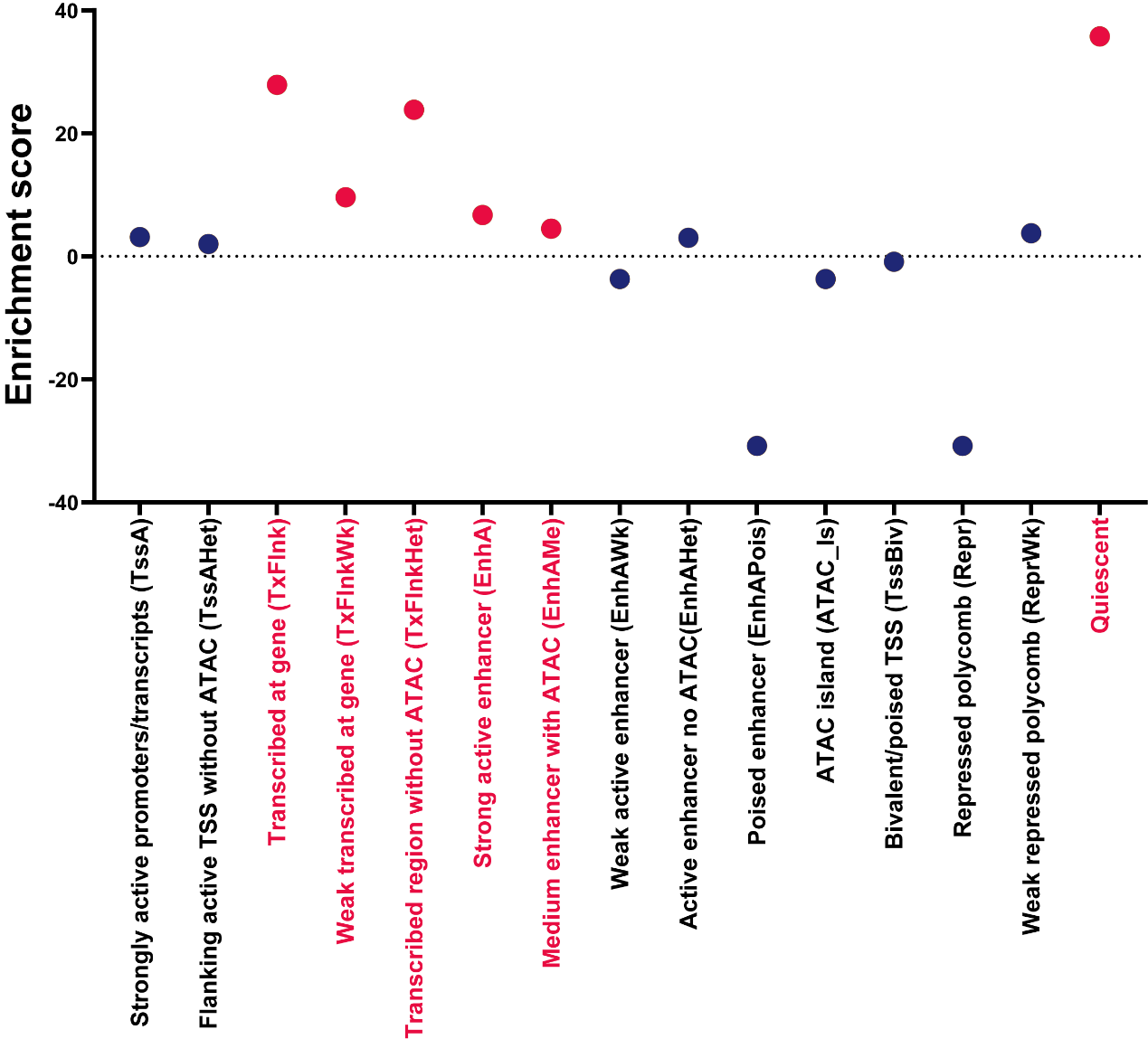


**Fig. S8** Enrichment of SNPs around immune core genes in 15 chromatin states. The red dots represent chromatin states where SNPs are significantly enriched


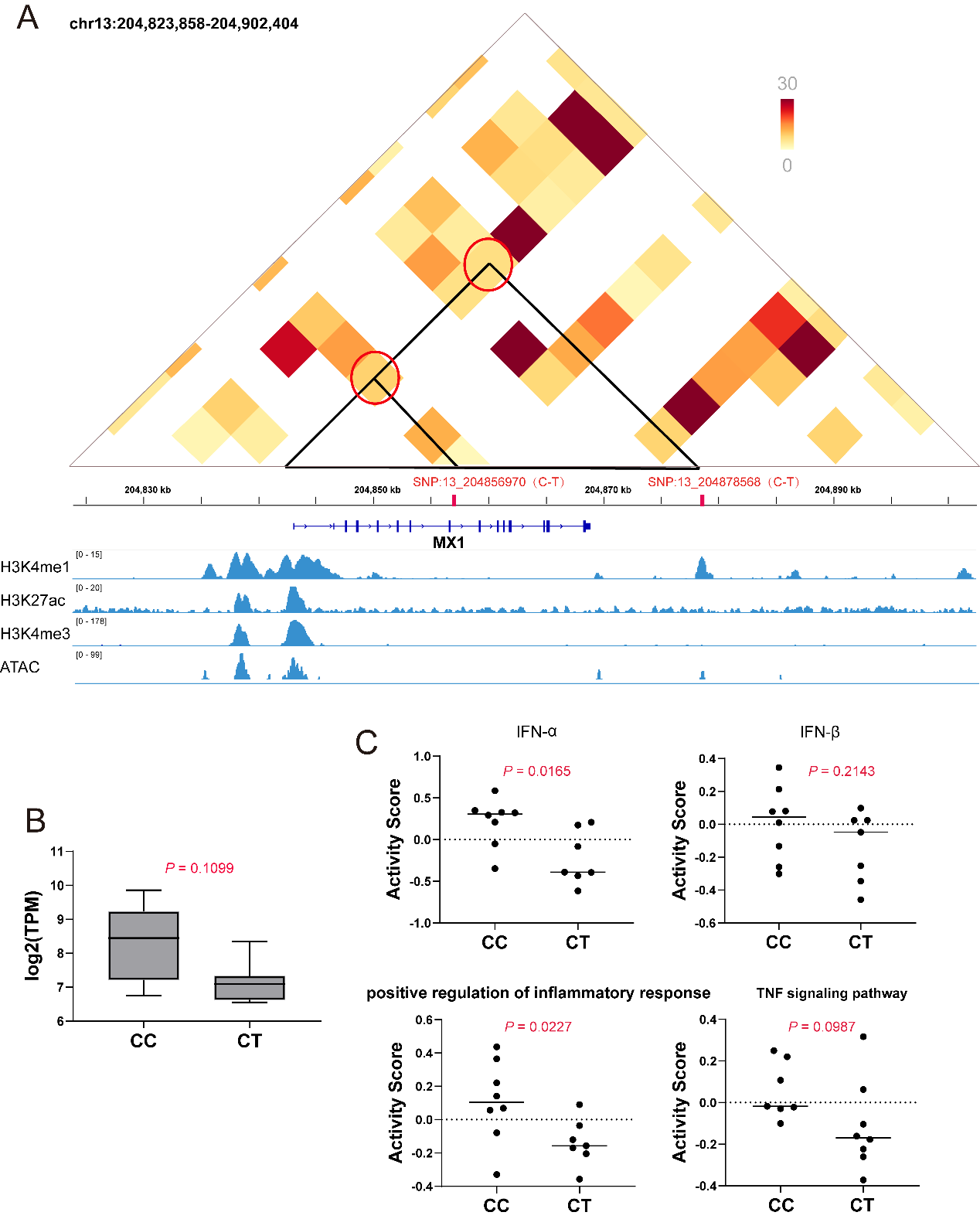


**Fig. S9** Two SNPs around *MX1* identified that are significantly associated with immunity. **A** Chromatin interaction and four epigenetic modifications around the *MX1* gene. **B** and **C** SNP:13_204856970(C-T)) is located in the gene body region of *MX1*, and the individuals with the C genotype of this SNP show higher gene expression and significantly higher immune pathway activity score


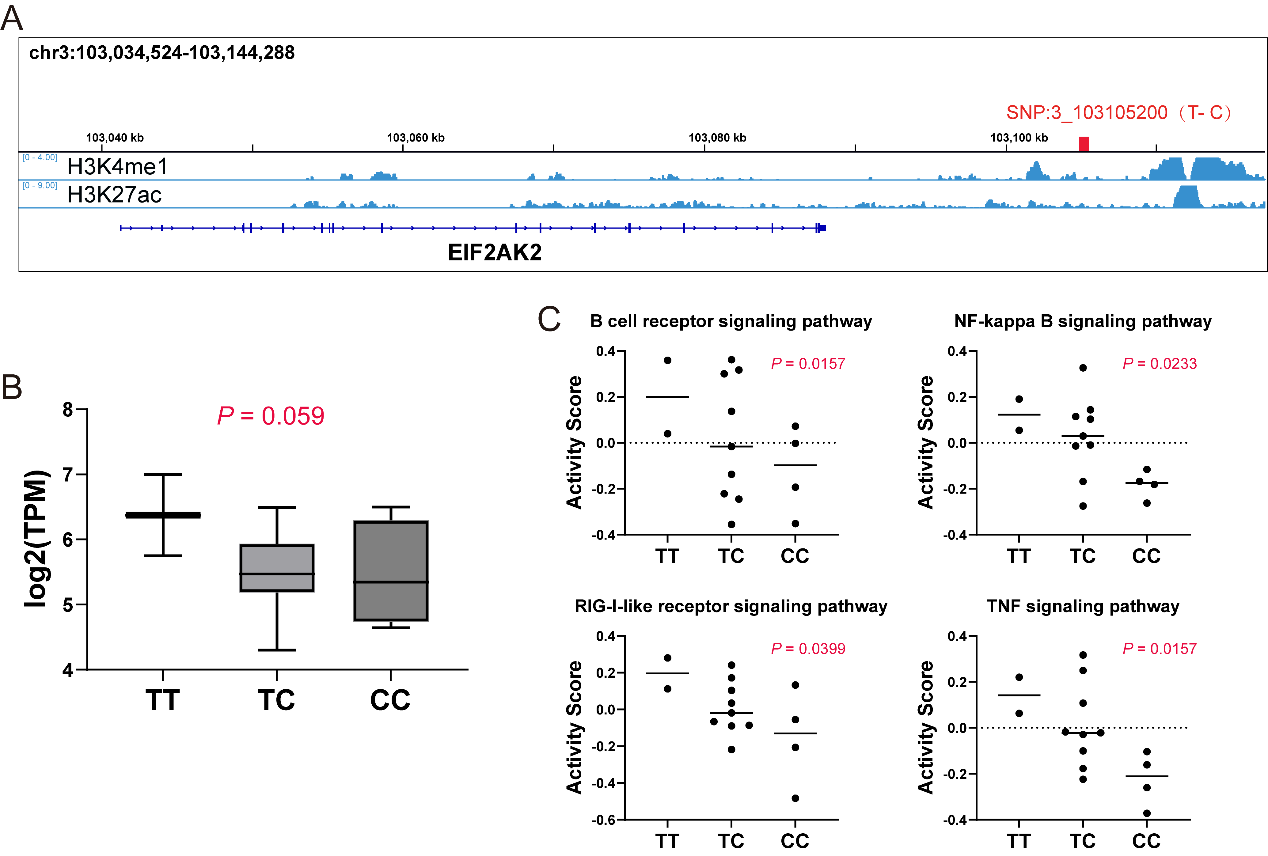


**Fig. S10** One SNP (3_103105200(T-C)) around *EIF2AK2* identified that was significantly associated with immunity. **A** Peak map of two epigenetic modifications around this SNP. **B** Gene expression of different genotypes of this SNP. **C** Immune pathway activity scores of different genotypes of this SNP


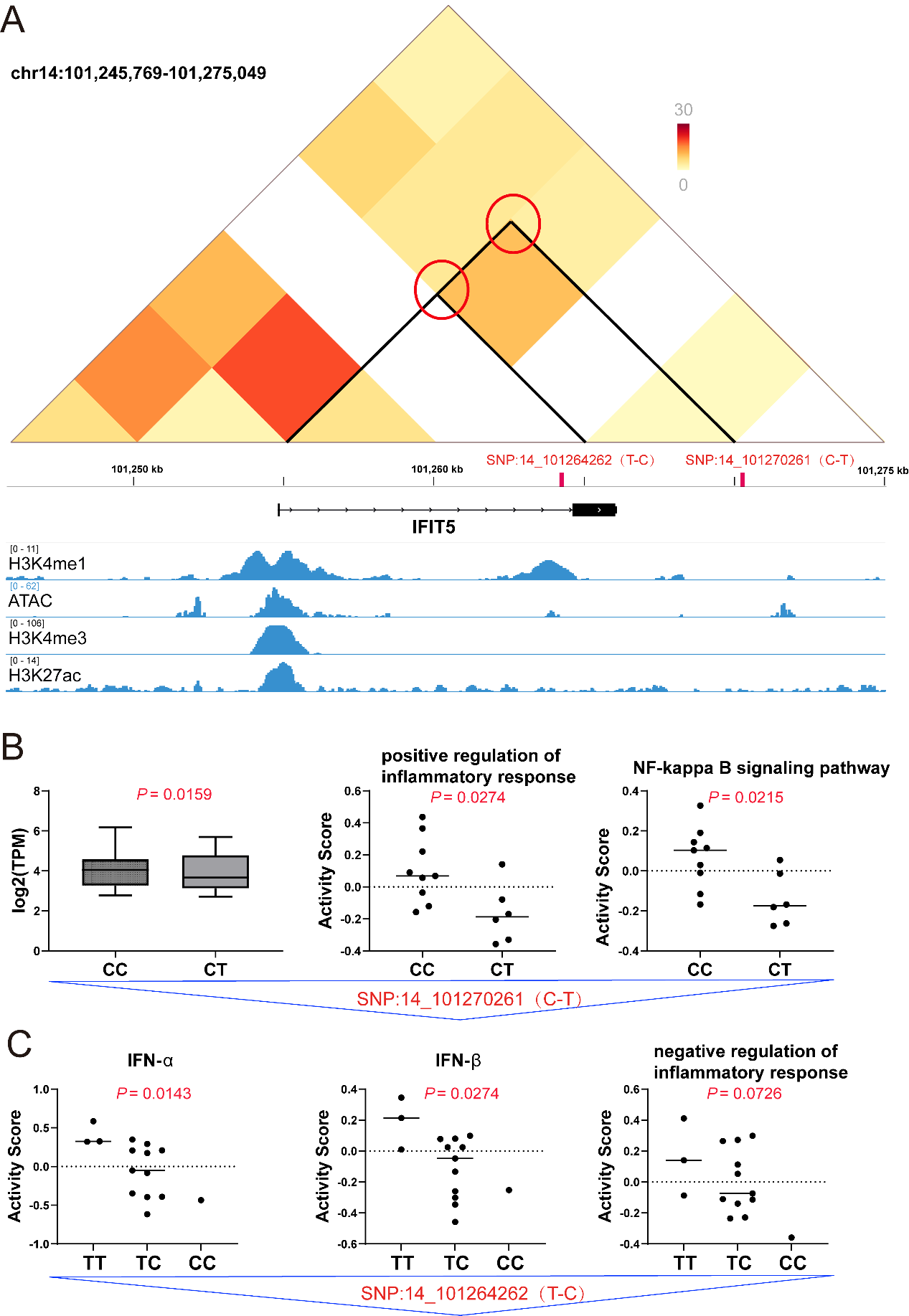


**Fig. S11** Two SNPs around *IFIT5* identified that are significantly associated with immunity. **A** Chromatin interaction and four epigenetic modifications around the *IFIT5* gene. **B** Gene expression and immune pathway activity scores of different genotypes of the SNP:14_101270261(C-T). **C** Immune pathway activity scores of different genotypes of the SNP:14_101264262(T-C)


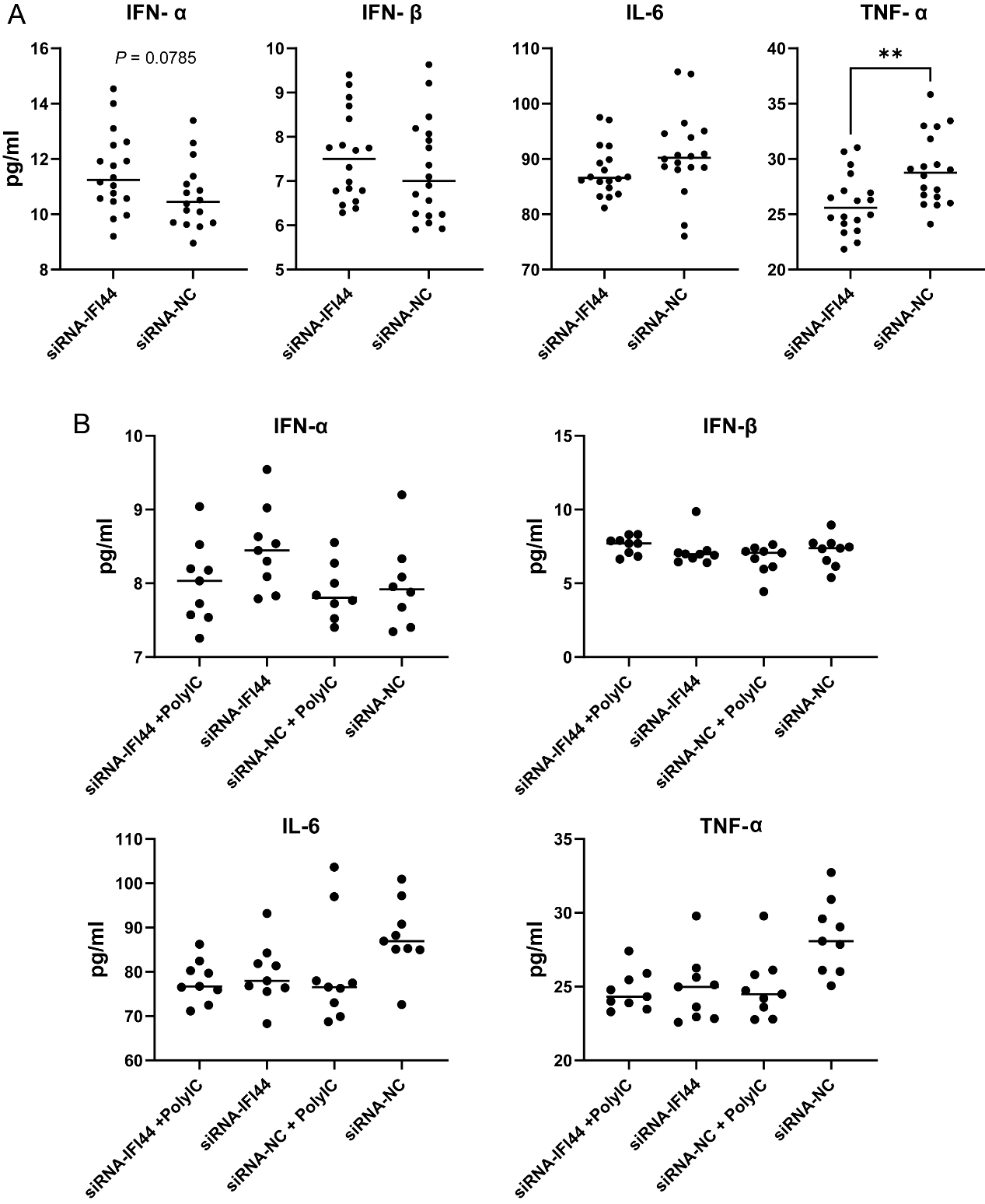


**Fig. S12** The effect of interfering with the *IFI44* gene on cytokine production. **A** The concentrations of 4 cytokines were detected after 24 hours of interference with *IFI44* gene. **B** After 24 h of interference with *IFI44* gene, Poly(I:C) was used for stimulation, and the concentrations of 4 cytokines were detected after another 24 h
